# Parabolic PDEs with Dynamic Data under a Bounded Slope Condition

**DOI:** 10.1007/s00205-026-02184-6

**Published:** 2026-04-12

**Authors:** Verena Bögelein, Frank Duzaar, Giulia Treu

**Affiliations:** 1 Fachbereich Mathematik, Universität Salzburg, Hellbrunner Str. 34, 5020 Salzburg, Austria; 2 Dipartimento di Matematica ‘Tullio Levi-Civita’, Universita’ di Padova, Via Trieste 63, 35121 Padua, Italy

## Abstract

We establish the existence of Lipschitz-continuous solutions to the Cauchy–Dirichlet problem for a class of evolutionary partial differential equations of the form

$$\begin{aligned} \partial _tu-{{\,\textrm{div}\,}}_x \nabla _\xi f(\nabla u)=0 \end{aligned}$$

in a space-time cylinder $\Omega _T=\Omega \times (0,T)$, subject to time-dependent boundary data $g:\partial _{\mathcal {P}}\Omega _T\rightarrow \mathbb {R}$ prescribed on the parabolic boundary. The main novelty in our analysis is a time-dependent version of the classical bounded slope condition, imposed on the boundary data *g* along the lateral boundary $\partial \Omega \times (0,T)$. More precisely, we require that, for each fixed $t\in [0,T)$, the graph of $g(\cdot ,t)$ over $\partial \Omega$ admits supporting hyperplanes with slopes that may vary in time but remain uniformly bounded. The key to handling time-dependent data lies in constructing more flexible upper and lower barriers.

## Introduction

Throughout this paper, let $\Omega \subset \mathbb {R}^n$, $n \ge 2$, denote a bounded, open, and convex set, and let $f :\mathbb {R}^n \rightarrow \mathbb {R}$ be a convex integrand. A classical theorem of Haar [24] ensures that, for prescribed boundary values $u_o :\partial \Omega \rightarrow \mathbb {R}$ satisfying the *bounded slope condition* (BSC), there exists a Lipschitz continuous minimizer $u :\Omega \rightarrow \mathbb {R}$ of the variational functional

1.1 $$\begin{aligned} \mathcal {F}(v) := \int _{\Omega } f(Dv) \,\textrm{d}x, \end{aligned}$$

subject to the boundary condition $u = u_o$ on $\partial \Omega$; see also [25, 26, 36, 40]. The construction has become standard and appears in modern textbooks on the calculus of variations; see, e.g., [22, Chapter 1]. More recent developments concerning the existence of Lipschitz minimizers under the BSC may be found in [10, 12, 13, 15, 31–34]. The standard argument hinges on a comparison principle in which the affine functions furnished by the BSC serve as barriers, together with the translation invariance of minimizers and a priori gradient bounds. The successful application of this strategy depends sensitively on the structure of the integrand, and in particular breaks down when lower-order terms are present. In such cases, affine functions no longer provide suitable barriers, and the method must be adapted by employing a more flexible class of barrier functions; see [19–21].

Surprisingly, a time-dependent counterpart to this semi-classical theory has remained an open problem for some time. Instead, sophisticated parabolic methods – such as Galerkin approximations, monotone operators, and nonlinear semigroup theory – have led to various existence results. However, the construction of Lipschitz continuous solutions to evolutionary equations associated with general convex integrands *f* has remained elusive without additional assumptions on the growth of the integrand. The paper [6] marks the first Haar-Rado-type result for the Cauchy-Dirichlet problem

$$\begin{aligned} \left\{ \begin{array}{cc} \partial _tu-{{\,\textrm{div}\,}}_x \nabla _\xi f(\nabla u)=0, &  \quad \text{ in } \Omega _T=\Omega \times (0,T),\\ u=u_o, &  \quad \text{ on } \partial _{\mathcal {P}}\Omega _T, \end{array} \right. \end{aligned}$$

which guarantees the existence of a unique classical solution $u\in C^0([0,T];L^2(\Omega )) \cap L^\infty (\Omega _T)$ with Lipschitz continuous spatial gradient $\nabla u\in L^\infty (\Omega _T,\mathbb {R}^n)$. The only conditions required are the convexity of the integrand $f:\mathbb {R}^n\rightarrow \mathbb {R}$, and the BSC imposed on the initial and lateral boundary datum $u_o\in W^{1,\infty }(\Omega )$. A related result for linear growth functionals can be found in [23]; see also [44]. The theory has subsequently been further generalized to data $u_o$ satisfying a one-sided BSC, as shown in [11, 37, 41]. A common feature of all these parabolic papers is the use of the BSC to construct affine barriers.

At this juncture, the natural question arises as to whether a semi-classical theory can be developed for time-dependent boundary values. Specifically, one might ask whether it is possible to construct Lipschitz solutions to the Cauchy-Dirichlet problem (1.3) with a boundary datum $g:\partial _{\mathcal {P}}\Omega _T\rightarrow \mathbb {R}$ depending on time. As we will discuss later, the proof strategy of [6] cannot, in principle, be applied to time-dependent boundary conditions. The barrier construction employed in [6] necessitates that the boundary values *g* be independent of time.

### Lipschitz Solutions

In the parabolic setting, the formulation of Lipschitz continuous variational solutions involves certain function spaces that can be interpreted as the parabolic analogue of Lipschitz continuous functions from the stationary setting. Specifically, we use the identification between the space of Lipschitz continuous functions, $\textrm{Lip}^x(\Omega ) = C^{0,1}(\Omega )$, and the Sobolev space $W^{1,\infty }(\Omega )$ to define the function space consisting of those bounded functions $v \in L^\infty (\Omega _T)$ having a bounded spatial gradient $\nabla v \in L^\infty (\Omega _T, \mathbb {R}^n)$, i.e.,

$$ \textrm{Lip}^x(\Omega _T) := \big \{ v\in L^{\infty }(\Omega _T):\nabla v\in L^\infty (\Omega _T,\mathbb {R}^n) \big \}. $$

For a given $L\in (0, \infty )$, we define the subclass $\textrm{Lip}^x(\Omega _T, L)$ as

$$ \textrm{Lip}^x(\Omega _T, L) := \big \{ v\in \textrm{Lip}^x(\Omega _T):\Vert \nabla v\Vert _{L^\infty (\Omega _T,\mathbb {R}^n)}\le L\big \}. $$

As a consequence of the identification of $\textrm{Lip}^x(\Omega _T)$ with $L^\infty (0,T, W^{1,\infty }(\Omega ))$, for almost every $t \in (0,T)$, the restriction $v(t):= v(\cdot , t)$ of $v \in \textrm{Lip}^x(\Omega _T)$ to the time slice $\Omega \times \{t\}$ belongs to $W^{1,\infty }(\Omega )$. This allows the classical definition of the trace on appropriate time slices. The space of Lipschitz continuous functions $\textrm{Lip}^x_0(\Omega _T)$ with zero lateral boundary values is also well-defined, enabling the formulation of the Cauchy-Dirichlet problem in the parabolic function space $g + \textrm{Lip}^x_0(\Omega _T)$ for some $g:\Omega _T\rightarrow \mathbb {R}$. This space consists of those functions $v \in \textrm{Lip}^x(\Omega _T)$ such that for almost every time slice $t \in (0,T)$ the restriction *v*(*t*) of *v* to $\Omega \times \{t\}$ belongs to $g(t) + W^{1,\infty }_0(\Omega ) \equiv g(t) + C^{0,1}_0(\Omega )$. For the boundary values *g*, we assume that

1.2 $$\begin{aligned} g \in \textrm{Lip}^x(\Omega _T)\text { with }\partial _t g \in L^2(\Omega _T),\text { and }g(0) := g_o \in W^{1,\infty }(\Omega ). \end{aligned}$$

Instead of $g+\textrm{Lip}^x_0(\Omega _T)$ we write $\textrm{Lip}^x_g(\Omega _T)$ for those $v\in \textrm{Lip}^x(\Omega _T)$ coinciding with *g* on the lateral boundary $\partial \Omega \times (0,T)$.

For the definition of the notion of *variational solution* to the parabolic Cauchy-Dirichlet problem

1.3 $$\begin{aligned} \left\{ \begin{array}{cc} \partial _tu-{{\,\textrm{div}\,}}_x \nabla _\xi f(\nabla u)=0, &  \quad \text{ in } \Omega _T=\Omega \times (0,T),\\ u=g, &  \quad \text{ on } \partial _{\mathcal {P}}\Omega _T \end{array} \right. \end{aligned}$$

we follow the approach of Lichnewsky and Temam [30], originally introduced in the context of the time-dependent parametric minimal surface equation. This idea leads naturally to the notion of variational solutions used in what follows.

#### Definition 1.1

*(Variational Solution)* Assume that the Cauchy-Dirichlet datum *g* fulfills hypothesis (1.2). A map $u\in \textrm{Lip}^x_g(\Omega _T)$ is called *variational solution on* $\Omega _{T}$ to the Cauchy-Dirichlet problem (1.3) if and only if the variational inequality

1.4 $$\begin{aligned} \iint _{\Omega _\tau }f(\nabla u)\,\textrm{d}x\textrm{d}t&\le \iint _{\Omega _\tau }\big [\partial _{t}v(v-u) + f(\nabla v)\big ]\,\textrm{d}x\textrm{d}t\nonumber \\&\quad + \tfrac{1}{2}\Vert v(0)-g_{o}\Vert _{L^{2}(\Omega )}^{2} - \tfrac{1}{2}\Vert (v-u)(\tau )\Vert _{L^{2}(\Omega )}^{2} \end{aligned}$$

holds true, for a.e. $\tau \in [0,T]$ and for any $v\in \textrm{Lip}^x_{g}(\Omega _T)$ with $\partial _{t}v\in L^{2}(\Omega _{T})$ and $v(0)\in L^2(\Omega )$.

Note that all terms in (1.4) are well-defined, since $u \in \textrm{Lip}^x_g(\Omega _T)$ implies $u \in L^\infty (0,T; L^2(\Omega ))$. By assumption (1.2), the function *g* is admissible as a test function in the variational inequality (1.4). This permits testing with $v = g$, which yields certain energy estimates. As a consequence, one can conclude that variational solutions satisfy the initial condition $u(\cdot , 0)=g_o$ in the usual $L^2(\Omega )$ sense, as demonstrated in Lemma 3.1. The concept of a variational solution enables the use of techniques from the calculus of variations. Under certain conditions, it can be demonstrated that a variational solution also satisfies the properties of a weak solution; see Theorem 1.4 below.

As in the classical theory of variational problems, we begin by constructing variational solutions subject to a *gradient constraint*. In this regard, we introduce a notion of variational solution that incorporates such a constraint. Assuming that for some $L> 0$, we have

1.5 $$\begin{aligned} L>\max \big \{ \Vert \nabla g_o\Vert _{L^\infty (\Omega ,\mathbb {R}^n)}, \Vert \nabla g\Vert _{L^\infty (\Omega _T,\mathbb {R}^n)}\big \}, \end{aligned}$$

the subclass $\textrm{Lip}^x_g(\Omega _T, L)$ is non-empty, and the following definition of variational solutions with gradient constraints is well-posed:

#### Definition 1.2

*(Variational solutions, gradient constraint)* Let *g* be as in (1.2) and $L>0$ as in (1.5). A map $u\in \textrm{Lip}^x_g(\Omega _T,L)$ is called *variational solution of the gradient constrained Cauchy-Dirichlet problem* (1.3) in $\textrm{Lip}^x_g(\Omega _T,L)$ if and only if the variational inequality

1.6 $$\begin{aligned} \iint _{\Omega _\tau }f(\nabla u)\textrm{d}x\textrm{d}t&\le \iint _{\Omega _\tau }\big [\partial _{t}v(v-u) + f(\nabla v)\big ]\textrm{d}x\textrm{d}t\nonumber \\&\quad + \tfrac{1}{2}\Vert v(0)-g_{o}\Vert _{L^{2}(\Omega )}^{2} - \tfrac{1}{2}\Vert (v-u)(\tau )\Vert _{L^{2}(\Omega )}^{2} \end{aligned}$$

holds true, for a.e. $\tau \in [0,T]$ and for any $v\in \textrm{Lip}^x_g(\Omega _T,L)$ with $\partial _{t}v\in L^{2}(\Omega _{T})$ and $v(0)\in L^2(\Omega )$. $\square$

### The Main Results

Our main result in this paper is the existence of Lipschitz continuous solutions (with respect to the spatial variable) to the Cauchy-Dirichlet problem (1.3) with time-dependent boundary values, under the assumption of the bounded slope condition. Before stating the main result, we first outline the *assumptions on the data*.

1. Let $\Omega \subset \mathbb {R}^n$ be *R*-uniformly convex for some $R>0$ in the sense of Definition 2.1;
2. Let $f:\mathbb {R}^n\rightarrow \mathbb {R}$ be convex, and, outside the unit ball $B_1=B_1(0)$, of class $C^{1,1}$ and uniformly convex. Specifically, there exists $\varepsilon \in (0,1]$, such that
  1.7 $$\begin{aligned} D^2f(\xi ) (\zeta , \zeta )\ge \varepsilon |\zeta |^2 \qquad \text{ for } \text{ a.e. } \xi \in \mathbb {R}^n\setminus B_1 \text{ and } \text{ any } \zeta \in \mathbb {R}^n; \end{aligned}$$
3. Let $g\in W^{1,\infty }(\Omega _T)$ be such that its restriction to the lateral boundary, $g|_{\partial \Omega \times [0,T]}$, satisfies the time-dependent bounded slope condition $t-\textrm{BSC}_Q$ in the sense of Definition 2.3, for some constant $Q>0$. In addition, assume that for each $x_o\in \partial \Omega$, the associated functions $w_{x_o}^\pm$ from the bounded slope condition are Lipschitz continuous in time, that is,
  $$ w_{x_o}^\pm \in W^{1,\infty }([0,T],\mathbb {R}^n), $$
  and that
  $$ \mathsf Q:= \sup _{x_o\in \partial \Omega }\Vert (w_{x_o}^\pm )'\Vert _{L^\infty ([0,T],\mathbb {R}^n)}<\infty . $$

Then the following existence result holds:

#### Theorem 1.3

(Existence of Lipschitz solutions) Suppose that assumptions (A1)–(A3) are satisfied. Then there exists a unique variational solution to the Cauchy–Dirichlet problem (1.3) in the sense of Definition 1.1  which satisfies the gradient bound

$$ \Vert \nabla u\Vert _{L^\infty (\Omega _T,\mathbb {R}^n)} \le C, $$

where *C* depends on $n, \varepsilon , R, {{\,\textrm{diam}\,}}(\Omega ), f, \nabla f, \Vert D^2f\Vert _{L^\infty (\mathbb {R}^n\setminus B_1)}, Q, [g]_{0,1;\Omega _T}$, and $\mathsf Q$.

We emphasize that the variational solution is unique even when the integrand *f* is convex but not strictly convex; see [9, Lemma 3.3]. This includes the case of integrands with flat regions. Moreover, if $f\in C^1$, then the variational solution constructed above enjoys additional regularity.

#### Theorem 1.4

(Regularity of solutions) Suppose that assumptions (A1)–(A3) are satisfied, and that $f\in C^1$. Then the variational solution *u* obtained in Theorem 1.3 is a weak solution to the Cauchy–Dirichlet problem (1.3), and satisfies $u\in C^{0;1,\frac{1}{2}}(\Omega _T)$.

### Novelty and Key Technical Tools

The proof proceeds in two steps. The first establishes the existence of variational solutions for time-dependent boundary data, under a uniform Lipschitz bound on the admissible class. This reduces to a variational inequality with a gradient constraint. The construction follows De Giorgi’s minimizing movement scheme, cf. [2], which is particularly suited to incorporating time-dependent Dirichlet data on the lateral boundary into the class of admissible competitors, cf. [5, 9, 38]. An alternative derivation, based on the method of weighted energy dissipation, cf. [1, 3, 4, 35], draws on De Giorgi’s variational framework for nonlinear evolution equations, including applications to certain nonlinear wave equations; see [17, 28, 39, 42]. This may be viewed as the first step in the spirit of Haar’s [24] approach to constructing Lipschitz minimizers of the parametric area functional–a perspective that anticipated modern developments in geometric analysis and regularity theory for minimal surfaces.

The solutions obtained are variational solutions in the sense of Lichnewsky and Temam [30]; that is, they solve a variational inequality subject to a gradient constraint of the form $|\nabla u|\le L$, where $L>0$ is a fixed constant. As a result, the class of admissible variations is restricted to those that preserve this structural bound. In particular, the presence of the constraint limits perturbations to directions that remain within the admissible set. It is therefore necessary to establish that the variational solution lies strictly inside the admissible class, in the sense that $\Vert \nabla u\Vert _{\infty }<L$; only then can arbitrary variations be performed, and the solution identified as the sole admissible one satisfying the variational inequality in the unconstrained class.

In the classical setting, this difficulty was addressed by Hilbert [27] through the introduction of the bounded slope condition, later refined by Haar [24] in the construction of Lipschitz minimizers of the parametric area functional. By restricting attention to graphs with uniformly bounded gradient, compactness is restored and direct methods become applicable, obviating the need for unconstrained variations. The bounded slope condition furnishes barriers required for comparison and maximum principles, and renders the explicit gradient constraint superfluous by enforcing uniform control through the prescribed bound.

The use of affine barriers derived from the bounded slope condition fundamentally relies on temporal constancy of the Dirichlet boundary data. This constraint becomes evident upon inspecting the argument in [6, Theorem 1.2], where for a fixed point $x_o\in \partial \Omega$ the function $g(x_o,t)+w_{x_o}^-(t)\cdot (x-x_o)$ is employed as a lower barrier and must act as a sub-solution to (1.3)$_1$. Differentiation in time yields the constraint

$$ \partial _t\big (g(x_o,t)+w_{x_o}^-(t)\cdot (x - x_o)\big ) = \partial _t g(x_o,t)+ \partial _t w_{x_o}^-(t)\cdot (x-x_o) \le 0 $$

for any $(x,t)\in \Omega _T$. Taking the limit $x\rightarrow x_o$ within $\Omega$ implies $\partial _t g(x_o,t)\le 0$ for any $t\in (0,T)$. A symmetric argument involving an upper barrier of the form $g(x_o,t)+w_{x_o}^+(t)\cdot (x-x_o)$ leads to the inequality $\partial _t g(x_o,t)\ge 0$. One is thus forced to conclude that *g* is stationary on the lateral boundary $\partial \Omega \times (0,T)$. In the presence of genuinely time-dependent boundary values, such affine constructions therefore fail to apply. While the bounded slope condition retains its structural role, the analysis in the presence of time-dependent boundary data necessitates more flexible constructions: the use of affine sub- or super-solutions, though formally admissible, inherently restricts the boundary values to be time-independent and is therefore incompatible with the temporal variability intrinsic to the problem.

Instead of employing affine barriers, we implement a construction based on the convex conjugate $f^*$, following an observation due to Cellina [14]. The central idea exploits the fact that, when the integrand *f* is of class $C^2$, a function of the form

$$\begin{aligned} v(x,t) = \frac{n}{\alpha }f^*\Big (\frac{\alpha }{n}\big (x-y(t)\big )\Big )-c(t), \end{aligned}$$

with $\alpha \in \mathbb {R}\setminus \{0\}$, $y:[0,T]\rightarrow \mathbb {R}^n$, and $c:[0,T]\rightarrow \mathbb {R}$ satisfies

$$\begin{aligned} {{\,\textrm{div}\,}}_x\nabla _\xi f\big (\nabla v(x,t)\big )=\alpha . \end{aligned}$$

In particular, if $$\alpha >0$$, then a choice of $$\alpha $$, *y*, and *c* such that $$\alpha $$ dominates the time derivative $$\partial _tv$$ at every point $$(x,t)\in \Omega _T$$ would ensure that *v* is a sub-solution to the parabolic equation (1.3), and thus a potential candidate for a barrier from below. However, being a subsolution is only one of the requirements for a function to qualify as a barrier from below. In addition, it must be compatible with the boundary data in the sense that, for some fixed boundary point $$x_o\in \partial \Omega $$, one has $$ v(x_o,t)=g(x_o,t)$$ for any $$t\in [0,T)$$, and $$v\le g$$ on $$\partial _{\mathcal {P}}\Omega _T$$. At this stage, the principal difficulty lies in ensuring that the real parameter $$\alpha $$, together with the functions *y* and *c*, can indeed be chosen so that *v* constitutes a lower barrier. This is realized by considering, for each $$t\in [0,T)$$, the set

$$\begin{aligned} \widetilde{\Omega }_t := \bigg \{x\in \mathbb {R}^n: \underbrace{\frac{n}{\alpha }f^*\Big (\frac{\alpha }{n}\big (x-y(t)\big )\Big )-c(t)}_{=\, v(x,t)}\le g(x_o,t)+\widetilde{w}_{x_o}^{-}(t)\cdot (x-x_o)\bigg \}, \end{aligned}$$

where $\widetilde{w}_{x_o}^{-}$ is a suitable modification of the affine function $w_{x_o}^{-}$, chosen to satisfy $g(x_o,t)+\widetilde{w}_{x_o}^{-}(t)\cdot (x-x_o)\le g(x,t)$ for all $(x,t)\in \Omega _T$. We then carefully select *y*(*t*) and *c*(*t*) such that, on the one hand,

$$\begin{aligned} x_o\in \partial \widetilde{\Omega }_t \quad \text{ for } \text{ all } t\in [0,T), \end{aligned}$$

and, on the other hand,

$$\begin{aligned} \Omega \subset \widetilde{\Omega }_t \quad \text{ for } \text{ all } t\in [0,T). \end{aligned}$$

By the definition of the sets $\widetilde{\Omega }_t$, this implies $v(x_o,t)=g(x_o,t)$ for all $t\in [0,T)$ and $v\le g$ in $\Omega _T$. Next, we establish an upper bound for $|\partial _t v|$ in $\Omega _T$ that is independent of $\alpha$. This enables us to choose $\alpha$ sufficiently large to guarantee that *v* is a subsolution. Finally, we compute an upper bound on the spatial gradient $\nabla v$, thereby demonstrating that the constructed lower barrier is Lipschitz continuous. Realizing this approach would already be delicate for $C^2$-integrands. The weaker assumption (A2) on *f* requires even greater care to balance the parameters involved and to avoid circular dependencies.

### Explicit Construction of a Lower Barrier in a Specific Example

To illustrate the construction of the lower barriers summarized in the preceding subsection and described in detail in § 6, we provide a simple example in which these barriers are explicitly constructed. Thereby, we use the notation introduced in § 1.3 and § 6. Let us consider the Cauchy-Dirichlet problem (1.3), where $\Omega =B_1(0)\subset \mathbb {R}^2$, $f(\xi )=\frac{1}{2} |\xi |^2$ with convex conjugate $f^*(\eta )=\frac{1}{2} |\eta |^2$, and boundary data $g(x,t)=x_1\cos t+x_2\sin t =(\cos t,\sin t)\cdot (x_1,x_2)$. We observe that, in this case, the boundary values are affine in $x=(x_1,x_2)$ for each fixed *t*, and they satisfy the $t-\textrm{BSC}_Q$ condition with constant $Q=1$; see Definition 2.3 below. In particular, we note that the $t-\textrm{BSC}_Q$ is satisfied for any $x_o\in \partial B_1(0)$ with the choice $w_{x_o}^-(t)=(\cos t,\sin t)$. In the setting of this example, the function defined in (6.1) takes the form

$$\begin{aligned} v(x,t)=\tfrac{\alpha }{4}|x-y(t)|^2-c(t), \end{aligned}$$

where $\alpha >0$ is a parameter, and $y(t)\in \mathbb {R}^2$, $c(t)\in \mathbb {R}$ are functions of time. We fix the point $x_o=(-1,0)\in \partial B_1(0)$ and aim to show that one can choose $\alpha$, *y*, and *c* such that the following conditions are satisfied:

1. $$v(x_o,t)=g(x_o,t)$$ for all $$t\in [0,T)$$;
2. $$v(x,t)\le g(x,t)$$ for all $$(x,t)\in \partial _{\mathcal {P}}\big (B_1(0)\times [0,T)\big )$$;
3. *v* is a sub-solution of
  1.8 $$\begin{aligned} \partial _t v-\Delta v =0 \qquad \text { in } B_1(0)\times [0,T). \end{aligned}$$

For $\alpha >0$ we now choose *y*(*t*) and *c*(*t*) such that (C1) and (C2) are satisfied. To this end, we define, cf. § 6,

$$\begin{aligned} \widetilde{\Omega }_t&= \big \{x\in \mathbb {R}^2: \tfrac{\alpha }{4}|x-y(t)|^2-c(t)\le g(x_o,t)+w_{x_o}^-(t)\cdot (x-x_o)\big \} \\&= \big \{x\in \mathbb {R}^2: \tfrac{\alpha }{4}|x-y(t)|^2-c(t)\le x_1 \cos t+x_2\sin t\big \}. \end{aligned}$$

Note that $g(x_o,t)+w_{x_o}^-(t)\cdot (x-x_o)= g(x,t)$ for any $(x,t)\in B_1(0)\times [0,T)$ and hence we can take $\widetilde{w}_{x_o}^-=w_{x_o}^-$ in Lemma 2.5. Next, we choose

$$ y(t)=(y_1(t),y_2(t))=\tfrac{2}{\alpha }\left( 1-\cos t,-\sin t\right) $$

and

$$ c(t)= \tfrac{\alpha }{4}+1+\tfrac{2}{\alpha }\left( 1-\cos t\right) , $$

and demonstrate that $x_o=(-1,0)\in \partial \widetilde{\Omega }_t$ and $B(0,1)\subset \widetilde{\Omega }_t$. Through direct calculations, we obtain

$$ \widetilde{\Omega }_t = B_{1+\frac{2}{\alpha }}\big (\tfrac{2}{\alpha },0\big )\quad \text{ for } \text{ every } t\in [0,T), $$

and hence $\widetilde{\Omega }_t$ satisfies the desired properties. To verify (C3), i.e. that *v*(*x*, *t*) is a subsolution of (1.8) for appropriate values of $\alpha >0$, we proceed as follows. By the properties of *f* and $f^*$ stated in Proposition 6.1, it immediately follows that $\Delta v={{\,\textrm{div}\,}}_x\nabla _\xi f(\nabla v)=\alpha$ in $B_1(0)\times [0,T)$. By a straightforward computation, we obtain for the time derivative of *v* that

$$\begin{aligned} \partial _t v(x,t)&=-x_1\sin t+x_2\cos t . \end{aligned}$$

We can conclude that *v* is a subsolution of (1.8) in the set $B_1(0)\times [0,T)$ for any $\alpha \ge 1$. Moreover, we estimate that $|\nabla v|\le 2+\frac{\alpha }{2}$ in $B_1(0)\times [0,T)$.

**Fig. 1:**
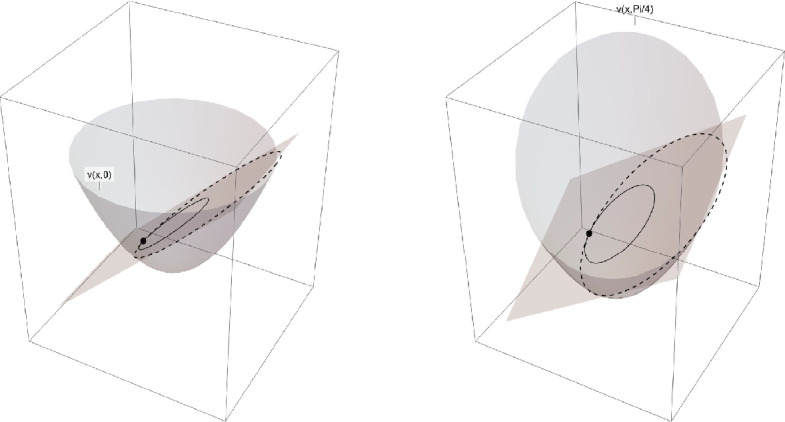
Construction for the value $\alpha =2$, at time $t=0$ on the left and at time $t=\frac{\pi }{4}$ on the right

In the figure (see Fig. 1) we represent the above construction for the fixed value $\alpha =2$, at time $t=0$ on the left and at time $t=\frac{\pi }{4}$ on the right. To be more precise, in the picture on the left there are the affine function involved in the Bounded Slope Condition for the point $x_o=(-1,0)$ and the corresponding barrier; the continuous line is the boundary condition, while the dashed line is the trace of the barrier function on $\partial \widetilde{\Omega }_0$. The picture on the right has to be interpreted analogously.

## Notations and Preliminaries

Throughout the paper, if not further specified, $\Omega$ denotes a bounded, open and convex subset of $\mathbb {R}^{n}$, $n\ge 2$ and $\Omega _T:=\Omega \times (0,T)$ is the associated space-time cylinder, $T>0$. The parabolic boundary of $\Omega _{T}$ is defined by

$$ \partial _{\mathcal {P}}\Omega _{T} := \left( \overline{\Omega }\times \{0\}\right) \cup \left( \partial \Omega \times (0,T)\right) . $$

As already mentioned, we frequently use the identification of the class of Lipschitz continuous functions $C^{0,1}(\overline{\Omega })$ with the space $W^{1,\infty }(\Omega )$. For a continuous function $u:\overline{\Omega }\rightarrow \mathbb {R}$ we recall the definition of the Lipschitz constant

$$ {[}u]_{0,1;\Omega }:=\sup _{x\not =y, x,y\in \Omega }\frac{|u(x)-u(y)|}{|x-y|}<\infty . $$

Since $\Omega$ is convex, we have that $[u]_{0,1;\Omega }=\Vert \nabla u\Vert _{L^\infty (\Omega ,\mathbb {R}^n)}$.

### *R*-Uniform Convexity and Time Dependent Bounded Slope Condition

In this subsection we specify the notion of *R*-uniform convexity and the time dependent bounded slope condition which have already been used in the statement of Theorem 1.3.

#### Definition 2.1

Let $R>0$. A subset $\Omega \subset \mathbb {R}^n$ is called *R*-*uniformly convex*, if for any $x_o\in \partial \Omega$ there exists a unit vector $\nu _{x_o}\in \mathbb {R}^n$ pointing outside of $\Omega$ such that

2.1 $$\begin{aligned} R\nu _{x_o}\cdot (x-x_o)\le - \tfrac{1}{2} |x-x_o|^2 \qquad \text{ for } \text{ any } x\in \partial \Omega . \end{aligned}$$

#### Remark 2.2

The notion of *R*-uniform convexity implies that for any $x\in \partial \Omega$ there holds

$$\begin{aligned} |x-(x_o-R\nu _{x_o})|^2&= |x-x_o|^2 +2R\nu _{x_o}\cdot (x-x_o)+R^2\le R^2. \end{aligned}$$

For the last inequality we used that $\nu _{x_o}$ points out of $\Omega$ and (2.1). In particular, we have $\Omega \subset B_R(x_o-R\nu _{x_o})$.

Since the boundary values *g* depend on time, we have to introduce a time dependent version of the bounded slope condition.

#### Definition 2.3

A function $g:\partial \Omega \times [0,T]\rightarrow \mathbb {R}$ satisfies the *time dependent bounded slope condition with constant* $Q>0$ (hereafter abbreviated as $t-\textrm{BSC}_Q$), if for each $x_o\in \partial \Omega$ there exist functions $w_{x_o}^\pm :[0,T]\rightarrow \mathbb {R}^n$ satisfying $\Vert w_{x_o}^{\pm }\Vert _{L^\infty ([0,T],\mathbb {R}^n)}\le Q$ such that

$$ g(x_o,t)+w_{x_o}^-(t)\cdot (x-x_o) \le g(x,t) \le g(x_o,t)+w_{x_o}^+(t)\cdot (x-x_o) $$

for any $$(x,t)\in \partial \Omega \times [0,T]$$.

#### Remark 2.4

In the framework of stationary problems Miranda [22, 36] proved that if $\Omega$ is a uniformly convex, bounded domain and $v\in C^2(\overline{\Omega })$, then $v|_{\partial \Omega }$ satisfies the bounded slope condition. In the time dependent setting it follows that if *g*(*x*, *t*) is a $C^{2}(\overline{\Omega _T})$ function, then it satisfies the $t-\textrm{BSC}_Q$ for some $Q>0$.

The time dependent bounded slope condition from Definition 2.3 involves a function *g* which is defined only on the lateral boundary of the parabolic cylinder. In the next lemma we will see an application for a function *G* defined on the whole parabolic cylinder and whose boundary values satisfy the $t-\textrm{BSC}_Q$.

#### Lemma 2.5

Let $G:\Omega \times [0,T]\rightarrow \mathbb {R}$ with $\sup _{t\in [0,T]}\textrm{Lip}^xG(\cdot ,t)\le Q_o$ such that $g:=G\big \vert _{\partial \Omega \times [0,T]}$ satisfies the $t-\textrm{BSC}_{Q}$ for some constant *Q*. Then for every $x_o\in \partial \Omega$ there exist $\widetilde{w}_{x_o}^\pm :[0,T]\rightarrow \mathbb {R}^n$ with $\Vert \widetilde{w}_{x_o}^\pm \Vert _{L^\infty ([0,T],\mathbb {R}^n)}\le Q + Q_o$ such that for any $(x,t)\in \overline{\Omega }\times [0,T]$ we have

$$\begin{aligned} g(x_o,t)+\widetilde{w}_{x_o}^{-}(t)\cdot (x-x_o) \le G(x,t) \le g(x_o,t)+\widetilde{w}_{x_o}^{+}(t)\cdot (x-x_o). \end{aligned}$$

If we additionally assume that $w_{x_o}^\pm \in W^{1,\infty }([0,T],\mathbb {R}^n)$, then

$$ \Vert (\widetilde{w}_{x_o}^\pm )'\Vert _{L^\infty ([0,T],\mathbb {R}^n)}=\Vert (w_{x_o}^\pm )'\Vert _{L^\infty ([0,T],\mathbb {R}^n)}. $$

#### Proof

Fix a boundary point $x_o\in \partial \Omega$ and denote by $[0,T]\ni t\mapsto w_{x_o}^{+}(t)$ the $\mathbb {R}^n$-valued function from the $t-\textrm{BSC}_{Q}$ satisfied by *g*. Recall that $|w_{x_o}^{+}(t)|\le Q$ and

$$\begin{aligned} g(x,t)\le g(x_o,t)+w_{x_o}^{+}(t)\cdot (x-x_o) \end{aligned}$$

for any $x\in \partial \Omega$ and any $t\in [0,T]$. Next, we fix a time $t\in [0,T]$. Without loss of generality we assume that $x_o=0$, that $g(x_o,t)=0$, and that $\Omega \subset \mathbb {R}^{n-1}\times \mathbb {R}_+$. Consider $x=(x',x_n)\in \Omega$. By $(x',y_n)\in \partial \Omega$ we denote the unique point in $\partial \Omega$ with $0\le y_n <x_n$. Then, we obtain

$$\begin{aligned} G(x,t)&\le G(x',x_n,t)-g(x',y_n,t)+ w_{x_o}^+(t)\cdot (x',y_n)\\&\le Q_o (x_n-y_n)+w_{x_o}^+(t)\cdot (x',y_n)\\&\le Q_o (x_n-y_n)+(w_{x_o}^+(t))'\cdot x'+(w_{x_o}^+(t))_n y_n\\&\le Q_o x_n+(w_{x_o}^+(t))'\cdot x'+|w_{x_o}^+(t))_n| x_n\\&\le \big [\underbrace{Q_oe_n+ \overline{w}_{x_o}^+(t)}_{=:\,\widetilde{w}_{x_o}^+(t)}\big ]\cdot (x',x_n), \end{aligned}$$

where we used that $0\le y_n<x_n$ and we defined $\overline{w}_{x_o}^+(t))=(w_{x_o}^+(t))',|w_{x_o}^+(t))_n|)$. Observing that $|\overline{w}_{x_o}^+(t))|=|w_{x_o}^+(t))|\le Q$ we have proven that *g* satisfies the $t-\textrm{BSC}_{Q_1}$ with the larger constant $Q_1=Q+Q_o$. $\square$

### Mollification in Time

In the definition of variational solutions we do not impose any condition on the time derivative. Therefore, in general we cannot insert the variational solution itself as comparison map in the variational inequality (1.4). Hence, a suitable mollification procedure in time is needed. For a separable Banach space *X*, an initial datum $v_{o}\in X$ and an integrability exponent $1\le r\le \infty$, we consider $v\in L^{r}(0,T;X)$ and $h\in (0,T]$ and define the mollification in time by

2.2 $$\begin{aligned} {[}v]_{h}(t):=\textrm{e}^{-\frac{t}{h}}v_{o} +\tfrac{1}{h}\int _{0}^{t}\textrm{e}^{\frac{s-t}{h}}v(s)\,\textrm{d}s, \end{aligned}$$

whenever $t\in [0,T]$. Later on, we use for instance $X=L^{r}(\Omega ,\mathbb {R}^{N})$ or $X=W^{1,r}(\Omega ,\mathbb {R}^{N})$ and the related parabolic spaces $L^{r}(0,T;L^{r}(\Omega ,\mathbb {R}^{N}))$, respectively $L^{r}(0,T;W^{1,r}(\Omega ,\mathbb {R}^{N}))$. One particular feature of this mollification is that $\left[ v\right] _{h}$ solves the ordinary differential equation

$$\begin{aligned} \partial _{t}[v]_{h}=\tfrac{1}{h}\big (v-[v]_{h}\big ) \end{aligned}$$

with initial condition $[v]_{h}(0)=v_{o}$. Note that, since $[v]_{h}$ solves the above ODE, then clearly any common membership of both *v* and its regularization $[v]_{h}$ to a Banach space is passed to the time derivative $\partial _t [v]_{h}$. The basic properties of the mollification in time are summarized in the following lemma (cf. [29, Lemma 2.2] and [3, Appendix B] for the proofs).

#### Lemma 2.6

Suppose *X* is a separable Banach space and $v_{o}\in X$. If $v\in L^{r}(0,T{;}X)$ for some $r\ge 1$, then also $[v]_{h}\in L^{r}(0,T;X)$, and $[v]_{h}\rightarrow v$ in $L^{r}\left( 0,T;X\right)$ as $h\downarrow 0$ if $r<\infty$. Furthermore, for any $t_{o}\in (0,T]$ it holds that

$$ \left\| [v]_{h}\right\| _{L^{r}\left( 0,t_{o};X\right) } \le \left\| v\right\| _{L^{r}\left( 0,t_{o};X\right) } + \left[ \tfrac{h}{r}\left( 1-\textrm{e}^{-\frac{t_{o}r}{h}}\right) \right] ^{\frac{1}{r}} \left\| v_{o}\right\| _{X}. $$

In the case $r=\infty$, the bracket $\left[ \ldots \right] ^{1/r}$ in the preceding inequality has to be interpreted as 1. Moreover, we have $\partial _{t}[v]_{h}\in L^{r}\left( 0,T;X\right)$ with

2.3 $$\begin{aligned} \partial _{t}[v]_{h}=\tfrac{1}{h}\big (v-[v]_{h}\big ). \end{aligned}$$

If in addition $\partial _{t}v\in L^{r}(0,T;X)$ and $v_o=v(0)$, then

$$ \partial _{t}[v]_{h}=\tfrac{1}{h}\int _{0}^{t}\textrm{e}^{\frac{s-t}{h}}\partial _{s}v(s)\textrm{d}s$$

and

$$ \left\| \partial _{t}[v]_{h}\right\| _{L^{r}\left( 0,T;X\right) } \le \left\| \partial _{t}v\right\| _{L^{r}\left( 0,T;X\right) }. $$

Finally, if $v\in C^{0}([0,T];X)$ and $v_o=v(0)$, then also $[v]_{h}\in C^{0}([0,T];X)$, $[v]_{h}(0)=v_{o}$ and $[v]_{h}\rightarrow v$ in $L^{\infty }([0,T];X)$ as $h\downarrow 0$. $\square$

## Properties of Variational Solutions

In this section we present some properties of variational solutions that are direct consequences of the definition. These properties can be achieved in a fairly general setting, in particular without assuming the bounded slope condition.

### Lemma 3.1

Assume that $f:\mathbb {R}^n\rightarrow \mathbb {R}$ is convex and bounded from below and *g* satisfies (1.2). Then, any variational solution *u* in the sense of Definition 1.1, resp. 1.2 fulfills the initial condition $u(0) = g_o$ in the $L^2$-sense, i.e. we have

$$ \lim _{h\downarrow 0} \tfrac{1}{h}\int _0^h\Vert (g-u)(\tau )\Vert _{L^{2}(\Omega )}^{2}\,\textrm{d}\tau =0. $$

### Proof

In view of assumption (1.2) we find $L>0$ satisfying (1.5). Since *g* is an admissible comparison map in (1.4), resp. (1.6), we have

$$\begin{aligned} \tfrac{1}{2}\Vert (g-u)(\tau )\Vert _{L^{2}(\Omega )}^{2} + \iint _{\Omega _\tau }f(\nabla u)\,\textrm{d}x\textrm{d}t&\le \iint _{\Omega _\tau }\big [\partial _{t}g(g-u) + f(\nabla g)\big ]\,\textrm{d}x\textrm{d}t\\&\le \iint _{\Omega _\tau }|\partial _{t}g||g-u|\,\textrm{d}x\textrm{d}t+ \tau |\Omega | \sup _{|\xi |\le L} f(\xi ) , \end{aligned}$$

for a.e. $\tau \in [0,T]$. On the left-hand side we discard the non-negative energy term. Then, we integrate for given $h>0$ with respect to $\tau$ over the interval [0, *h*]. In this way we get

$$\begin{aligned} \tfrac{1}{2}\int _0^h&\Vert (g-u)(\tau )\Vert _{L^{2}(\Omega )}^{2}\,\textrm{d}\tau \\&\le h\iint _{\Omega _h}|\partial _{t}g||g-u|\,\textrm{d}x\textrm{d}t+ \tfrac{1}{2} h^2|\Omega | \bigg [\sup _{|\xi |\le L} f(\xi ) + \Big |\inf _{\mathbb {R}^n} f\Big |\bigg ]. \end{aligned}$$

We divide both sides by *h* and apply Young’s inequality to the first term on the right. This gives

$$\begin{aligned} \tfrac{1}{h}\int _0^h&\Vert (g-u)(\tau )\Vert _{L^{2}(\Omega )}^{2}\,\textrm{d}\tau \\&\le \iint _{\Omega _h} \big [|\partial _{t}g|^2+|g-u|^2\big ]\, \textrm{d}x\textrm{d}t+ h|\Omega | \bigg [\sup _{|\xi |\le L} f(\xi ) + \Big |\inf _{\mathbb {R}^n} f\Big |\bigg ]. \end{aligned}$$

Note that $\partial _tg\in L^2(\Omega _T)$ implies $g\in C^0( [0,T];L^2(\Omega ))$. As the right-hand side vanishes in the limit $h\downarrow 0$ and $g(0)=g_o$, the claim follows. $\square$

In the next lemma we show that variational solutions in the sense of Definition 1.1 apriori are of class $C^0( [0,T]; L^2(\Omega ))$. The argument is similar to [9, Lemma 3.2]. Here, however, we have to estimate the difference of energy terms in a different way.

### Lemma 3.2

Assume that $f:\mathbb {R}^n\rightarrow \mathbb {R}$ is convex and *g* satisfies (1.2). Then, every variational solution *u* in the sense of Definition 1.1 satisfies

$$ u\in C^0\big ( [0,T]; L^2(\Omega )\big ). $$

### Proof

We test the variational inequality for *u* with

$$\begin{aligned} v_h= g+[u-g]_h =g+\tfrac{1}{h}\int _0^t\mathrm e^\frac{s-t}{h}\big (u(s)-g(s)\big )\,\textrm{d}s, \end{aligned}$$

where $[u-g]_h$ is defined as in (2.2) with initial value $v_o=0$. In view of Lemma 2.6, one easily shows $v_h\in \textrm{Lip}^x_{g}(\Omega _T)$, $\partial _t v_h\in L^2(\Omega _T)$, and $v_h(0)=g_o\in L^2(\Omega )$, so that $v_h$ is admissible in (1.4). In particular, we have a uniform bound for the spatial gradient $|\nabla v_h|$ in $\Omega _T$. In fact, using the definition of $v_h$ and assumption (1.2) we get

$$\begin{aligned} |\nabla v_h|&= \bigg |\nabla g +\tfrac{1}{h}\int _0^t\mathrm e^\frac{s-t}{h}\big (\nabla u(s)-\nabla g(s)\big )\,\textrm{d}s\bigg |\\&\le L+ \big (\Vert \nabla u\Vert _{L^\infty (\Omega _T,\mathbb {R}^n)}{+}L\big ) \tfrac{1}{h}\int _0^t\mathrm e^\frac{s-t}{h}\textrm{d}s\\&\le 2L+ \Vert \nabla u\Vert _{L^\infty (\Omega _T,\mathbb {R}^n)}=:\widetilde{L}. \end{aligned}$$

Testing the variational inequality (1.4) with $v_h$ we obtain

$$\begin{aligned} \tfrac{1}{2}\Vert (v_h-u)(\tau )\Vert _{L^{2}(\Omega )}^{2}&\le \iint _{\Omega _\tau }\partial _{t}v_h(v_h-u)\,\textrm{d}x\textrm{d}t+ \iint _{\Omega _\tau } \big [f(\nabla v_h)- f(\nabla u)\big ]\,\textrm{d}x\textrm{d}t\end{aligned}$$

for a.e. $\tau \in [0,T]$. In the last displayed inequality we pass to the supremum over $\tau \in (0,T)$. This implies

$$\begin{aligned} \tfrac{1}{2}\sup _{\tau \in (0,T)}&\Vert (v_h-u)(\tau )\Vert _{L^{2}(\Omega )}^{2}\\&\le \sup _{\tau \in (0,T)} \iint _{\Omega _\tau }\partial _{t}v_h(v_h-u)\,\textrm{d}x\textrm{d}t+ \iint _{\Omega _T} \big | f(\nabla v_h)- f(\nabla u)\big |\,\textrm{d}x\textrm{d}t. \end{aligned}$$

The second term on the right-hand side can be bounded by means of the gradient bound for $|\nabla v_h|$ in $\Omega _T$. Indeed, we get

$$\begin{aligned} \big | f(\nabla v_h)- f(\nabla u)\big |&\le \sup _{|\xi |\le \widetilde{L}} |\nabla f(\xi )| | \nabla v_h-\nabla u| \rightarrow 0\quad \text{ in } L^1(\Omega _T) \text{ as } h\downarrow 0, \end{aligned}$$

so that

$$\begin{aligned} \lim _{h\downarrow 0}\iint _{\Omega _T} \big | f(\nabla v_h)- f(\nabla u)\big |\,\textrm{d}x\textrm{d}t=0. \end{aligned}$$

Moreover, exploiting (2.3), we infer that

$$\begin{aligned}&\iint _{\Omega _\tau }\partial _{t}v_h(v_h-u)\,\textrm{d}x\textrm{d}t\\&\qquad = \iint _{\Omega _\tau }\Big [\partial _{t}g\big (g-u+[u-g]_h\big ) + \partial _{t}[u-g]_h \big (g-u+[u-g]_h\big )\Big ] \,\textrm{d}x\textrm{d}t\\&\qquad \le \iint _{\Omega _\tau } |\partial _{t}g| \big |u-g-[u-g]_h\big | \,\textrm{d}x\textrm{d}t. \end{aligned}$$

so that

$$\begin{aligned} \limsup _{h\downarrow 0} \sup _{\tau \in (0,T)} \iint _{\Omega _\tau }\partial _{t}v_h(v_h-u)\textrm{d}x\textrm{d}t\le 0, \end{aligned}$$

Inserting the preceding estimates above, we arrive at

$$\begin{aligned} \lim _{h\downarrow 0} \Vert v_h-u\Vert _{L^\infty (0,T; L^2(\Omega ))}=0. \end{aligned}$$

Note that $g\in C^0( [0,T];L^2(\Omega ))$, since $\partial _tg\in L^2(\Omega _T)$. This however means that also $v_h$ belongs to $C^0( [0,T];L^2(\Omega ))$. Thus $u\in L^\infty (0,T; L^2(\Omega ))$ is the limit with respect to $L^\infty ( 0,T; L^2(\Omega ))$-convergence of functions $v_h$ which are themselves of class $C^0( [0,T];L^2(\Omega ))$. As a result the limit map *u* is continuous with respect to time, i.e. $u\in C^0( [0,T];L^2(\Omega ))$. This proves the claim. $\square$

## Existence of Solutions for Regularized Boundary Data

In this section we establish for sufficiently regular boundary data *g* the existence of a variational solution to the gradient constrained problem in the sense of Definition 1.2 via the method of *minimizing movements* (finite time discretization). We assume that $g\in \textrm{Lip}^x(\Omega _T)$ additionally satisfies

4.1 $$\begin{aligned} \partial _tg\in L^2(\Omega _T)\cap L^\infty \big ( 0,T; W^{1,\infty }(\Omega )\big ) \text{ and } g_o:=g(0)\in W^{1,\infty }(\Omega ). \end{aligned}$$

This implies in particular that $g\in C^0( [0,T], L^2(\Omega ))$. Furthermore, let $L>0$ such that

4.2 $$\begin{aligned} \Vert \nabla g\Vert _{L^\infty (\Omega _T,\mathbb {R}^n)} < L. \end{aligned}$$

### Proposition 4.1

Let $f:\mathbb {R}^n\rightarrow \mathbb {R}$ be convex, $L>0$ and suppose that $g\in \textrm{Lip}^x(\Omega _T)$ satisfies (4.1) and (4.2). Then, there exists a unique variational solution *u* in $\Omega _T$ in the class $\textrm{Lip}^x_g(\Omega _T,L)$ in the sense of Definition 1.2. Moreover, we have $\partial _t u\in L^2(\Omega _T)$.

The proof of Proposition 4.1 will be established in § 4.1–4.3.

### A Sequence of Minimizers for Variational Functionals

We fix a step size $h\in (0,1]$ and define for $i\in \mathbb {N}_0$ with $ih\le T$ the time-discretized boundary values $g_i:=g(ih)\in W^{1,\infty } (\Omega )$. Note that $\Vert \nabla g_i\Vert _{L^\infty (\Omega ,\mathbb {R}^n)}\le L$. Our goal is to inductively construct a sequence $u_i\in g_i + W^{1,\infty }_0(\Omega )$ of Lipschitz minimizers to certain elliptic variational functionals satisfying the gradient constraint $\Vert \nabla u_i\Vert _{L^\infty (\Omega ,\mathbb {R}^n)}\le L$. The precise construction is as follows. Suppose that $u_{i-1}\in g_{i-1}+ W^{1,\infty }_0(\Omega )$ for some $i\in \mathbb {N}$ has already been defined. If $i=1$, then $u_0=g_o$ is the initial boundary datum. Then, we let $u_i$ be the minimizer of the variational functional

$$ \mathsf F_{i}[v] := \int _{\Omega }f(\nabla v)\,\textrm{d}x+\tfrac{1}{2h}\int _\Omega |v-u_{i-1}|^2\,\textrm{d}x\, . $$

in the class of functions $v\in g_i+ W^{1,\infty }_0(\Omega )$ with $\Vert \nabla v\Vert _{L^\infty (\Omega ,\mathbb {R}^n)}\le L$. Observe that this class in non-empty since $v=g_i$ is admissible. Note also that $\textsf{F}_{i}$ is bounded on this function class. The existence of a unique minimizer $u_i$ can be deduced by means of standard compactness arguments (i.e. by use of the Arzela-Ascoli theorem) using the convexity of *f*. We note that $\Vert \nabla u_i\Vert _{L^\infty (\Omega ,\mathbb {R}^n)}\le L$ for any $i\in \mathbb {N}_0$ with $ih\le T$, by construction.

Next, we want to compare the energy of $u_i$ with the one of $u_{i-1}$. This is not directly possible because the boundary values of $u_{i-1}$ do not coincide with those of $u_i$. We compensate for this by choosing as comparison function $u_{i-1}+g_i-g_{i-1}$ instead of $u_{i-1}$. From

$$ \mathsf F_{i}[u_i]\le \mathsf F_{i}[u_{i-1}+g_i-g_{i-1}] $$

we obtain

$$\begin{aligned} \int _{\Omega }f(\nabla u_i)\,\textrm{d}x&+ \tfrac{1}{2h}\int _\Omega |u_i-u_{i-1}|^2\,\textrm{d}x\\&\le \int _{\Omega }f\big (\nabla (u_{i-1}+g_i-g_{i-1})\big )\,\textrm{d}x+\tfrac{1}{2h}\int _\Omega |g_i-g_{i-1}|^2\,\textrm{d}x\\&= \int _{\Omega }f(\nabla u_{i-1})\,\textrm{d}x+ \textbf{I}_i+\textbf{II}_i, \end{aligned}$$

where

$$\begin{aligned} \textbf{I}_i&:= \tfrac{1}{2h}\int _\Omega |g_i-g_{i-1}|^2\,\textrm{d}x,\\ \textbf{II}_i&:= \int _{\Omega }\big [f\big (\nabla (u_{i-1}+g_i-g_{i-1})\big )-f(\nabla u_{i-1})\big ]\,\textrm{d}x. \end{aligned}$$

For $\textbf{I}_i$ we have

$$\begin{aligned} \textbf{I}_i&\le \tfrac{1}{2h}\int _\Omega \bigg |\int _{(i-1)h}^{ih} \partial _tg\,\textrm{d}t\bigg |^2\textrm{d}x\le \tfrac{1}{2}\iint _{\Omega \times [(i-1)h,ih]}|\partial _tg|^2\,\textrm{d}x\textrm{d}t. \end{aligned}$$

Similarly, we obtain

$$ \big |\nabla (g_i-g_{i-1})\big | \le \bigg |\int _{(i-1)h}^{ih} \partial _t\nabla g\,\textrm{d}t\bigg | \le h\Vert \partial _t\nabla g\Vert _{L^\infty (\Omega _T,\mathbb {R}^n)}, $$

so that

$$ \big \Vert \nabla (u_{i-1}+g_i-g_{i-1})\big \Vert _{L^\infty (\Omega )} \le L+ h\Vert \partial _t\nabla g\Vert _{L^\infty (\Omega _T,\mathbb {R}^n)}. $$

Note that the right-hand side is finite due to assumption (4.1). The preceding inequality can be used to estimate the integral $\textbf{II}_i$. in fact, we get

$$\begin{aligned} |\textbf{II}_i|&\le \int _{\Omega }\big |f\big (\nabla (u_{i-1}+g_i-g_{i-1})\big )-f(\nabla u_{i-1})\big |\,\textrm{d}x\\&\le \sup _{|\xi |\le L+ \Vert \partial _t\nabla g\Vert _{L^\infty (\Omega _T,\mathbb {R}^n)}} |\nabla f(\xi )| \int _\Omega |\nabla (g_i-g_{i-1})|\,\textrm{d}x\\&\le \mathsf K\iint _{\Omega \times [(i-1)h,ih]}|\partial _t\nabla g|\,\textrm{d}x\textrm{d}t, \end{aligned}$$

where

4.3 $$\begin{aligned} \mathsf K := \sup _{|\xi |\le L+ \Vert \partial _t\nabla g\Vert _{L^\infty (\Omega _T,\mathbb {R}^n)}} |\nabla f(\xi )|. \end{aligned}$$

Substituting the estimates for $\textbf{I}_i$ and $\textbf{II}_i$ gives

4.4 $$\begin{aligned} \nonumber&\int _{\Omega }f(\nabla u_i)\,\textrm{d}x+ \tfrac{1}{2h}\int _\Omega |u_i-u_{i-1}|^2\,\textrm{d}x\\&\qquad \le \int _{\Omega }f(\nabla u_{i-1})\,\textrm{d}x+ \iint _{\Omega \times [(i-1)h,ih]} \big [\tfrac{1}{2}|\partial _tg|^2 + K |\partial _t\nabla g|\big ]\,\textrm{d}x\textrm{d}t. \end{aligned}$$

By inductively comparing the energy of $u_i$ with that of $u_{i-1}$ for $i\in \{ 0,\dots ,\ell \}$ in the described way and summing with respect to *i*, we get for any $\ell \in \mathbb {N}$ with $\ell h\le T$ the *energy estimate*

4.5 $$\begin{aligned}&\int _\Omega f(\nabla u_\ell )\,\textrm{d}x+ \tfrac{1}{2h}\sum _{i=1}^\ell \int _\Omega |u_i-u_{i-1}|^2\,\textrm{d}x\nonumber \\&\qquad \le \int _\Omega f(\nabla g_{o})\,\textrm{d}x+ \iint _{\Omega _T}\big [\tfrac{1}{2}|\partial _tg|^2 + K|\partial _t\nabla g| \big ]\,\textrm{d}x\textrm{d}t. \end{aligned}$$

The first term of the right-hand side actually contains no new information, because due to the construction of the minimizers $u_\ell$ the $W^{1,\infty }(\Omega )$-norm of $u_\ell$ is bounded anyway. Indeed, for any $\ell$ as above we have

4.6 $$\begin{aligned} \Vert u_\ell \Vert _{L^\infty (\Omega )}+ \Vert \nabla u_\ell \Vert _{L^\infty (\Omega ,\mathbb {R}^n)}&\le \Vert g_\ell \Vert _{L^\infty (\Omega )}+ \Vert u_\ell -g_\ell \Vert _{L^\infty (\Omega )} + L\nonumber \\&\le \Vert g\Vert _{L^\infty (\Omega _T)}+ {{\,\textrm{diam}\,}}(\Omega )\Vert \nabla u_\ell -\nabla g_\ell \Vert _{L^\infty (\Omega )} +L\nonumber \\&\le \Vert g\Vert _{L^\infty (\Omega _T)}+L\big [ 1+2 {{\,\textrm{diam}\,}}(\Omega )\big ] =: L_1. \end{aligned}$$

From now on we consider only such values $h_\ell \in (0,1]$ which satisfy $\ell := \frac{T}{h_\ell }\in \mathbb {N}$. Then we define functions $u^{(h_\ell )}:\Omega \times (-h_\ell ,T]\rightarrow \mathbb {R}$ and $g^{(h_\ell )}:\Omega \times (-h_\ell ,T]\rightarrow \mathbb {R}$ by

$$\begin{aligned} u^{(h_\ell )}(\cdot , t):= u_i \text{ and } g^{(h_\ell )}(\cdot , t):= g_i \quad \text{ for } t\in \big ((i-1)h_\ell , ih_\ell \big ] \text{ with } i\in \{ 0,\dots ,\ell \}. \end{aligned}$$

Note that both $u^{(h_\ell )}$ and $g^{(h_\ell )}$ are piecewise constant with respect to time and that $u^{(h_\ell )}(t)=g^{(h_\ell )}(t)$ on $\partial \Omega$ for any $t\in (0,T)$ in the usual sense of traces of continuous maps on $\overline{\Omega }$. This is true since both $u^{(h_\ell )}(t)$ and $g^{(h_\ell )}(t)$ can be uniquely extended to $\overline{\Omega }$. From (4.6) we conclude that

4.7 $$\begin{aligned} \sup _{t\in [0,T]}\Big [ \big \Vert u^{(h_\ell )}\big \Vert _{L^\infty (\Omega )} + \big \Vert \nabla u^{(h_\ell )}\big \Vert _{L^\infty (\Omega ,\mathbb {R}^n)}\Big ]&\le L_1. \end{aligned}$$

Thus, there exist a map

$$ u\in \bigcap _{q\ge 1}L^{q}(0,T;W^{1,q}(\Omega )) $$

and a subsequence – still denoted by $(u^{(h_\ell )})_{\ell \in \mathbb {N}}$ – such that

4.8 $$\begin{aligned} \left\{ \begin{array}{cl} u^{(h_\ell )}\rightharpoondown u &  \text{ weakly } \text{ in } L^{q}(\Omega _{T}) \text{ for } \text{ any } q\ge 1,\\ \nabla u^{(h_\ell )}\rightharpoondown \nabla u &  \text{ weakly } \text{ in } L^{q}(\Omega _{T},\mathbb {R}^n) \text{ for } \text{ any } q\ge 1. \end{array}\right. \end{aligned}$$

Observe that by lower-semicontinuity we have for any $q\ge 1$ that4.9so that $\Vert \nabla u\Vert _{L^\infty (\Omega _T,\mathbb {R}^n)}\le L$. In particular, we have $u\in L^\infty ( 0,T;W^{1,\infty }(\Omega ))$.

So far we have no information about the time derivative of the limit map *u*. This can be achieved by exploiting the estimate for the second term on the left-hand side of (4.5). In fact, this term can be translated into an estimate of the time derivative of the function $\widetilde{u}^{(h_\ell )}:\Omega \times (-h_\ell , T]\rightarrow \mathbb {R}$, which is constructed by linear interpolation in time of the minimizing functions $u_{i-1}$ and $u_i$ on the time interval $( (i-1)h_\ell , ih_\ell ]$. More precisely, we define

$$ \widetilde{u}^{(h_\ell )}(\cdot , t) = \Big ( i-\frac{t}{h_\ell }\Big )u_{i-1} + \Big (1- i+\frac{t}{h_\ell }\Big )u_{i} \quad \text { for } t\in \big ( (i-1)h_\ell , ih_\ell \big ] $$

whenever $i\in \{ 0,\dots ,\ell \}$. Obviously, $u^{(h_\ell )}$ and $\widetilde{u}^{(h_\ell )}$ coincide on $\Omega \times (-h_\ell , 0]$ by definition. For $t\in ( (i-1)h_\ell , ih_\ell ]$ the time derivative of $\widetilde{u}^{(h_\ell )}$ can easily be computed as

$$ \partial _t\widetilde{u}^{(h_\ell )}(\cdot ,t)=\tfrac{1}{h_\ell } (u_i-u_{i-1}). $$

We use this to rewrite the second term on the left-hand side of (4.5). This yields

4.10 $$\begin{aligned} \tfrac{1}{2}\iint _{\Omega _T}\big |\partial _t\widetilde{u}^{(h_\ell )}\big |^2\,\textrm{d}x\textrm{d}t&\le \int _\Omega f(\nabla g_{o})\,\textrm{d}x+ \iint _{\Omega _T}\big [\tfrac{1}{2}|\partial _tg|^2 + \mathsf K|\partial _t\nabla g| \big ]\,\textrm{d}x\textrm{d}t\nonumber \\&\le |\Omega | \sup _{|\xi |\le L} |f(\xi )| + \iint _{\Omega _T}\big [\tfrac{1}{2}|\partial _tg|^2 + \mathsf K|\partial _t\nabla g| \big ]\,\textrm{d}x\textrm{d}t. \end{aligned}$$

Analogous to (4.7) we have

4.11 $$\begin{aligned} \sup _{t\in [0,T]}\Big [ \big \Vert \widetilde{u}^{(h_\ell )} \big \Vert _{L^\infty (\Omega )} + \big \Vert D\widetilde{u}^{(h_\ell )}\big \Vert _{L^\infty (\Omega ,\mathbb {R}^n)}\Big ]&\le L_1 . \end{aligned}$$

Thus, the sequence $\widetilde{u}^{(h_\ell )}$ is uniformly bounded in $L^\infty ( 0,T; W^{1,\infty }(\Omega ))$. In addition, the sequence of time derivatives $\partial _t\widetilde{u}^{(h_\ell )}$ is uniformly bounded in $L^2(\Omega _T)$. Together these imply that the sequence $(\widetilde{u}^{(h_\ell )})_{\ell \in \mathbb {N}}$ is uniformly bounded in $W^{1,2}(\Omega _T)$. By compactness, there exists a subsequence – still denoted by $(\widetilde{u}^{(h_\ell )})_{\ell \in \mathbb {N}}$ – and a limit function $\widetilde{u}\in \bigcap _{q\ge 1} L^q(0,T;W^{1,q}(\Omega ))$ with $\partial _t\widetilde{u}\in L^2(\Omega _T)$, such that in the limit $\ell \rightarrow \infty$ we have

4.12 $$\begin{aligned} \left\{ \begin{array}{cl} \widetilde{u}^{(h_\ell )}\rightarrow \widetilde{u} &  \quad \text{ strongly } \text{ in } L^{2}(\Omega _T),\\ \widetilde{u}^{(h_\ell )}\rightharpoondown \widetilde{u} &  \quad \text{ weakly } \text{ in } L^q\big (0,T;W^{1,q}(\Omega )\big ) \text{ for } \text{ any } q\ge 1, \\ \partial _t\widetilde{u}^{(h_\ell )}\rightharpoondown \partial _t\widetilde{u} &  \quad \text{ weakly } \text{ in } L^2(\Omega _T). \end{array} \right. \end{aligned}$$

As a first consequence of the weak $L^2$-convergence of the time derivative $(4.12)_3$ and the lower-semicontinuity of the $L^2$-norm we conclude from (4.10) that

$$\begin{aligned} \iint _{\Omega _T}|\partial _t\widetilde{u}|^2\,\textrm{d}x\textrm{d}t&\le \liminf _{\ell \rightarrow \infty } \iint _{\Omega _T}\big |\partial _t\widetilde{u}^{(h_\ell )}\big |^2\,\textrm{d}x\textrm{d}t\\&\le 2|\Omega | \sup _{|\xi |\le L} |f(\xi )| + \iint _{\Omega _T}\big [|\partial _tg|^2 + 2\mathsf K|\partial _t\nabla g| \big ]\,\textrm{d}x\textrm{d}t. \end{aligned}$$

In the further course of the proof we will see that $\widetilde{u}=u$, so that *u* has a time derivative in $L^2(\Omega _T)$ with the quantitative estimate

4.13 $$\begin{aligned} \iint _{\Omega _T}|\partial _t u|^2 \,\textrm{d}x\textrm{d}t&\le 2|\Omega | \sup _{|\xi |\le L} |f(\xi )| + \iint _{\Omega _T}\big [|\partial _tg|^2 + 2\mathsf K|\partial _t\nabla g| \big ]\,\textrm{d}x\textrm{d}t. \end{aligned}$$

Now we establish that $\widetilde{u}= u$. By comparing the two sequences $u^{(h_\ell )}$ and $\widetilde{u}^{(h_\ell )}$, it is easy to see that their limits coincide. Indeed, we have

$$ \big |\widetilde{u}^{(h_\ell )}-u^{(h_\ell )}\big | \le |u_i-u_{i-1}| \quad \text{ for } t\in \big ((i-1)h_\ell , ih_\ell \big ], $$

which together with (4.5) leads us to

$$\begin{aligned} \iint _{\Omega _T}\big |\widetilde{u}^{(h_\ell )}-u^{(h_\ell )}\big |^2\,\textrm{d}x\textrm{d}t&\le h_\ell \sum _{i=1}^\ell \int _\Omega |u_i-u_{i-1}|^2\,\textrm{d}x\\&\le h_\ell ^2\bigg [ 2|\Omega | \sup _{|\xi |\le L} |f(\xi )| + \iint _{\Omega _T}\big [|\partial _tg|^2 + 2 \mathsf K|\partial _t\nabla g| \big ]\,\textrm{d}x\textrm{d}t\bigg ]. \end{aligned}$$

Taking into account (4.12)$_1$, we obtain that $u^{(h_\ell )}\rightarrow \widetilde{u}$ strongly in $L^2(\Omega _T)$ as $\ell \rightarrow \infty$. Therefore, we have $\widetilde{u}=u$. The strong $L^2(\Omega _T)$-convergence allows us to pass to another (not relabelled) subsequence, which then converges almost everywhere, i.e. $u^{(h_\ell )}\rightarrow u$ a.e. in $\Omega _T$ as $\ell \rightarrow \infty$.

Now we turn our attention to the initial time $t=0$. With (4.12)$_1$ and (4.10) we obtain for $t\in (0,T)$ that

$$\begin{aligned} \frac{1}{t}\iint _{\Omega \times [0,t]}&|u(\tau )-g_o|^2\,\textrm{d}x\textrm{d}\tau \\&= \lim _{\ell \rightarrow \infty }\frac{1}{t}\iint _{\Omega \times [0,t]}\big |\widetilde{u}^{(h_\ell )}(\tau )-g_o\big |^2\,\textrm{d}x\textrm{d}\tau \\&= \lim _{\ell \rightarrow \infty }\frac{1}{t}\iint _{\Omega \times [0,t]} \bigg |\int _0^\tau \partial _\tau \widetilde{u}^{(h_\ell )}(s)\,\textrm{d}s\bigg |^2\,\textrm{d}x\textrm{d}\tau \\&\le \lim _{\ell \rightarrow \infty }\frac{1}{t}\iint _{\Omega \times [0,t]} \tau \int _0^\tau \big |\partial _\tau \widetilde{u}^{(h_\ell )}\big |^2\,\textrm{d}s\textrm{d}x\textrm{d}\tau \\&\le \bigg [ 2|\Omega | \sup _{|\xi |\le L} |f(\xi )| + \iint _{\Omega _T}\big [|\partial _tg|^2 + 2\mathsf K|\partial _t\nabla g| \big ]\,\textrm{d}x\textrm{d}t\bigg ] \frac{1}{t} \int _0^t\tau \,\textrm{d}\tau \\&= t \bigg [ |\Omega | \sup _{|\xi |\le L} |f(\xi )| + \iint _{\Omega _T}\big [\tfrac{1}{2}|\partial _tg|^2 + \mathsf K|\partial _t\nabla g| \big ]\,\textrm{d}x\textrm{d}t\bigg ]. \end{aligned}$$

This implies

$$\begin{aligned} \lim _{t\downarrow 0}\frac{1}{t}\iint _{\Omega \times [0,t]}|u(\tau )-g_o|^2\,\textrm{d}x\textrm{d}\tau = 0. \end{aligned}$$

### Minimizing Properties of the Approximation Sequences

In this subsection we show that the piecewise constant functions $u^{(h_\ell )}$ constructed via time discretization minimize a certain integral functional on a space-time cylinder. In fact, for fixed $\tau \in [0,T]$ and $\ell \in \mathbb {N}$ the function $u^{(h_\ell )}$ minimizes

$$ \mathsf F^{(h_\ell )}[v] := \iint _{\Omega _\tau } \Big [f\big (\nabla u^{(h_\ell )}\big ) + \tfrac{1}{2h_\ell }\big |v(t)-u^{(h_\ell )}(t-h_\ell )\big |^2 \Big ]\,\textrm{d}x\textrm{d}t$$

in the class of functions

4.14 $$\begin{aligned} v\in g^{(h_\ell )}+L^\infty \big (0,\tau ;W^{1,\infty }_{0}(\Omega )\big ) \text{ with } \displaystyle \Vert Dv\Vert _{L^\infty (\Omega _\tau )} \le L. \end{aligned}$$

This results from a simple calculation, using the definition of $u^{(h_\ell )}$, the minimizing property of $u_i$, and the definition of the functional $\textsf{F}^{(h_\ell )}$. Indeed, denoting by $\boldsymbol{\chi }_{[0,\tau ]}$ the characteristic function of the interval $[0,\tau ]$, we have

$$\begin{aligned} \mathsf F^{(h_\ell )}\big [u^{(h_\ell )}\big ]&= \sum _{i=1}^{\ell } \int _{(i-1)h_\ell }^{ih_\ell } \boldsymbol{\chi }_{[0,\tau ]} \int _{\Omega } \Big [\tfrac{1}{2h_\ell }|u_i-u_{i-1}|^2 + f(\nabla u_i)\Big ] \,\textrm{d}x\textrm{d}t\\&= \sum _{i=1}^{\ell } \int _{(i-1)h_\ell }^{ih_\ell } \boldsymbol{\chi }_{[0,\tau ]}\mathsf F_i[u_i] \,\textrm{d}t\le \sum _{i=1}^{\ell } \int _{(i-1)h_\ell }^{ih_\ell } \boldsymbol{\chi }_{[0,\tau ]}\mathsf F_i[v(t)] \,\textrm{d}t\\&= \sum _{i=1}^{\ell } \int _{(i-1)h_\ell }^{ih_\ell } \chi _{[0,\tau ]} \int _{\Omega } \Big [ f(\nabla v(t)) + \tfrac{1}{2h_\ell } \big |v(t)-u^{(h_\ell )}(t-h_\ell )\big |^2\Big ]\,\textrm{d}x\textrm{d}t\\&= \mathsf F^{(h_\ell )}[v]. \end{aligned}$$

We use this minimality condition below to derive a variational inequality for the approximating functions $u^{(h_\ell )}$. In this discrete variational inequality we can then proceed to the limit $\ell \rightarrow \infty$ and obtain that *u* is the desired solution. The precise procedure is as follows. We first re-write the above inequality in terms of the total energy of $u^{(h_\ell )}$ as follows

$$\begin{aligned} \iint _{\Omega _\tau }&f\big (\nabla u^{(h_\ell )}\big ) \,\textrm{d}x\textrm{d}t\\&\le \iint _{\Omega _\tau }f(\nabla v) \,\textrm{d}x\textrm{d}t\\&\phantom {\le \,}+ \tfrac{1}{2h_\ell } \iint _{\Omega _\tau } \Big [\big |v(t)-u^{(h_\ell )}(t{-}h_\ell )\big |^2 -\big |u^{(h_\ell )}(t)- u^{(h_\ell )}(t{-}h_\ell )\big |^2\Big ]\,\textrm{d}x\textrm{d}t\\&= \iint _{\Omega _\tau }f(\nabla v) \,\textrm{d}x\textrm{d}t\\&\phantom {\le \,}+ \tfrac{1}{h_\ell } \iint _{\Omega _\tau } \Big [\tfrac{1}{2}\big |v-u^{(h_\ell )}\big |^2 + \big (v-u^{(h_\ell )}\big )\big (u^{(h_\ell )}-u^{(h_\ell )}(t{-}h_\ell )\big )\Big ]\,\textrm{d}x\textrm{d}t. \end{aligned}$$

We now replace *v* by the convex combination of $u^{(h_\ell )}$ and a general admissible function *v* as in (4.14), i.e. the function $w^{(h_\ell )}:=u^{(h_\ell )}+s(v-u^{(h_\ell )})$ with $s\in (0,1)$. Note that $w^{(h_\ell )}$ is still admissible, because $w^{(h_\ell )}$ fulfills both requirements in (4.14). Using also the convexity of *f*, we obtain

$$\begin{aligned}&\iint _{\Omega _\tau } f\big (\nabla u^{(h_\ell )} \big ) \,\textrm{d}x\textrm{d}t\\&\qquad \le \iint _{\Omega _\tau } f\big (\nabla u^{(h_\ell )}+s(\nabla v-\nabla u^{(h_\ell )}) \big ) \,\textrm{d}x\textrm{d}t\\&\qquad \phantom {=\ }+ \tfrac{1}{h_\ell } \iint _{\Omega _T} \Big [\tfrac{s^2}{2}\big |v-u^{(h_\ell )}\big |^2 + s\big (v-u^{(h_\ell )}\big )\big (u^{(h_\ell )}-u^{(h_\ell )}(t-h_\ell )\big )\Big ] \,\textrm{d}x\textrm{d}t\\&\qquad \le \iint _{\Omega _\tau } \Big [(1-s)f\big (\nabla u^{(h_\ell )}\big ) + sf(\nabla v)\Big ] \,\textrm{d}t\\&\qquad \phantom {=\ }+ \tfrac{1}{h_\ell }\iint _{\Omega _\tau } \Big [\tfrac{s^2}{2}\big |v-u^{(h_\ell )}\big |^2 + s\big (v-u^{(h_\ell )}\big )\big (u^{(h_\ell )}-u^{(h_\ell )}(t-h_\ell )\big )\Big ]\,\textrm{d}x\textrm{d}t. \end{aligned}$$

Here we absorb the first integral on the right-hand side into the left. Then we divide by $s>0$ and let $s\downarrow 0$. This yields

$$\begin{aligned} \iint _{\Omega _\tau } f\big (\nabla u^{(h_\ell )} \big ) \,\textrm{d}x\textrm{d}t&\le \iint _{\Omega _\tau } f(\nabla v)\,\textrm{d}x\textrm{d}t+ \iint _{\Omega _\tau } \big (v-u^{(h_\ell )}\big )\Delta _{-h_\ell } u^{(h_\ell )}\,\textrm{d}x\textrm{d}t, \end{aligned}$$

where

$$ \Delta _{-h_\ell } u^{(h_\ell )} := \tfrac{1}{h_\ell }\big (u^{(h_\ell )}-u^{(h_\ell )}(t-h_\ell )\big ). $$

In the second integral on the left-hand side we perform a discrete integration by parts. For this, however, it is necessary that *v* is also defined for negative times. Therefore, we define $v(t):=v(0)$ for $t<0$ and assume that $v(0)\in L^2(\Omega )$ exists. This allows us to conclude

4.15 $$\begin{aligned} \nonumber \iint _{\Omega _\tau }&f\big (\nabla u^{(h_\ell )} \big )\,\textrm{d}x\textrm{d}t\\\nonumber&\le \iint _{\Omega _\tau }f(\nabla v) \,\textrm{d}x\textrm{d}t+ \tfrac{1}{h_\ell } \iint _{\Omega _\tau } \big (v-u^{(h_\ell )}\big )\big (v-v(t-h_\ell )\big ) \,\textrm{d}x\textrm{d}t\\\nonumber&\quad + \tfrac{1}{2h_\ell } \iint _{\Omega _\tau } \Big [\big |v-u^{(h_\ell )}\big |^2(t-h_\ell ) - \big |v-u^{(h_\ell )}\big |^2\Big ] \,\textrm{d}x\textrm{d}t\\\nonumber&\quad - \tfrac{1}{2h_\ell } \iint _{\Omega _\tau } \big |v - v(t-h_\ell ) - u^{(h_\ell )} + u^{(h_\ell )}(t-h_\ell )\big |^2 \,\textrm{d}x\textrm{d}t\\\nonumber&\le \iint _{\Omega _\tau }f(\nabla v) \,\textrm{d}x\textrm{d}t+ \iint _{\Omega _\tau } \big (v-u^{(h_\ell )}\big )\Delta _{-h_\ell }v \,\textrm{d}x\textrm{d}t\\&\phantom {=\ }- \tfrac{1}{2h_\ell } \iint _{\Omega \times [\tau -h_\ell ,\tau ]} \big |v-u^{(h_\ell )}\big |^2 \,\textrm{d}x\textrm{d}t+ \tfrac{1}{2h_\ell } \iint _{\Omega \times [-h_\ell ,0]} |v-g_o|^2 \,\textrm{d}x\textrm{d}t\,. \end{aligned}$$

### Variational Inequality for the Limit Map

Our aim here is to perform the limit $h_\ell \downarrow 0$ in (4.15). To this aim we need to replace the boundary condition $v = g^{(h_\ell )}$ on the lateral boundary by the $h_\ell$-independent condition $v = g$. Therefore we consider $v\in \textrm{Lip}^x_g(\Omega _T,L)$ satisfying $\partial _tv\in L^2(\Omega _T)$ and $v(0)\in L^2( \Omega )$. Moreover, we extend *v* to negative times $t<0$ by $v(t):=v( 0)\in L^2(\Omega )$. In (4.15) we would like to insert $v+g^{(h_\ell )}-g$ as comparison map. However, this is not possible due to the gradient constraint. This problem can be compensated by considering a convex combination of *v* and *g* instead of *v*. More precisely, for $\lambda \in (0,1)$, we consider

$$ v^{(h_\ell )} := \lambda g +(1-\lambda )v+g^{(h_\ell )}-g $$

as a comparison map. Note that $v^{(h_\ell )}\in g^{(h_\ell )} +L^\infty (0,T;W^{1,\infty }_0(\Omega ))$. Moreover, in view of (4.2) we have

$$\begin{aligned} \Vert \nabla (\lambda g +(1-\lambda )v)\Vert _{L^\infty (\Omega _T,\mathbb {R}^n)} \le \lambda \Vert \nabla g\Vert _{L^\infty (\Omega _T,\mathbb {R}^n)} + (1-\lambda ) \Vert \nabla v\Vert _{L^\infty (\Omega _T,\mathbb {R}^n)} {<} L, \end{aligned}$$

and also

$$\begin{aligned} \big \Vert \nabla g^{(h_\ell )}-\nabla g\big \Vert _{L^\infty (\Omega _T,\mathbb {R}^n)}&\le h_\ell \Vert \partial _t\nabla g\Vert _{L^\infty (\Omega _T,\mathbb {R}^n)}. \end{aligned}$$

Therefore, for $\ell \in \mathbb {N}$ large enough, i.e. for $\ell$ with

$$ h_\ell \Vert \partial _t\nabla g\Vert _{L^\infty (\Omega _T,\mathbb {R}^n)} \le L- \Vert \nabla \big (\lambda g+ (1-\lambda )v\big )\Vert _{L^\infty (\Omega _T,\mathbb {R}^n)}, $$

the function $v^{(h_\ell )}$ fulfills the gradient constraint, so that $v^{(h_\ell )}\in \textrm{Lip}^x_g(\Omega _T,L)$ and hence it is admissible in (4.15). In the sequel we consider the individual terms on the right-hand side of (4.15). We start with the integral involving the integrand *f*. The comparison of the energies of $v^{(h_\ell )}$ and $\lambda g +(1-\lambda )v$ shows

$$\begin{aligned} \iint _{\Omega _\tau }&\big | f\big (\nabla v^{(h_\ell )}\big )- f\big ( \nabla (\lambda g +(1-\lambda )v)\big )\big |\,\textrm{d}x\textrm{d}t\\&\le \sup _{|\xi |\le L+ \Vert \partial _t\nabla g\Vert _{L^\infty (\Omega _T,\mathbb {R}^n)}}|\nabla f(\xi )| \iint _{\Omega _\tau }\big | \nabla g^{(h_\ell )}-\nabla g\big |\,\textrm{d}x\textrm{d}t\\&\le \mathsf K h_\ell \iint _{\Omega _T}| \partial _t \nabla g|\,\textrm{d}x\textrm{d}t, \end{aligned}$$

for any $\tau \in [0,T]$, where $\mathsf K$ is defined in (4.3). From this inequality and the convexity of *f* we obtain

4.16 $$\begin{aligned} \iint _{\Omega _\tau }&f\big (\nabla v^{(h_\ell )}\big )\,\textrm{d}x\textrm{d}t\nonumber \\&\le \iint _{\Omega _\tau } f\big ( \nabla (\lambda g +(1-\lambda )v)\big )\,\textrm{d}x\textrm{d}t+ \mathsf K h_\ell \iint _{\Omega _T}| \partial _t \nabla g|\,\textrm{d}x\textrm{d}t\nonumber \\&\le \lambda \iint _{\Omega _\tau } f(\nabla g)\,\textrm{d}x\textrm{d}t+ (1-\lambda ) \iint _{\Omega _\tau } f(\nabla v)\,\textrm{d}x\textrm{d}t+ \mathsf K h_\ell \iint _{\Omega _T}| \partial _t \nabla g|\,\textrm{d}x\textrm{d}t. \end{aligned}$$

Next we consider the term involving the time derivative, i.e the second integral on the right-hand side of (4.15), and observe that

$$\begin{aligned} \Delta _{-h_\ell } v^{(h_\ell )} \rightarrow \lambda \partial _t g +(1-\lambda )\partial _t v \quad \text{ strongly } \text{ in } L^2(\Omega _T), \end{aligned}$$

since $\partial _tv, \partial _tg\in L^2(\Omega _T)$ by assumption. Together with (4.8)$_1$ (here we only need the conclusion for $q=2$), this implies

4.17 $$\begin{aligned} \nonumber \lim _{\ell \rightarrow \infty } \iint _{\Omega _\tau }&\big (v^{(h_\ell )}-u^{(h_\ell )}\big )\Delta _{-h_\ell }v^{(h_\ell )} \,\textrm{d}x\textrm{d}t\\&= \lambda \iint _{\Omega _\tau }(v-u) \partial _t g \,\textrm{d}x\textrm{d}t+ (1-\lambda )\iint _{\Omega _\tau }(v-u) \partial _tv\,\textrm{d}x\textrm{d}t. \end{aligned}$$

Next, we turn our attention to the last two integrals on the right-hand side of (4.15). Using $v^{(h_\ell )}(t)=\lambda g_o+(1-\lambda ) v( 0)$ for $t\in (-h_\ell ,0)$, the last integral in (4.15) takes the form

4.18 $$\begin{aligned} \tfrac{1}{2h_\ell } \iint _{\Omega \times [-h_\ell ,0]}\big |v^{(h_\ell )}-g_o\big |^2 \,\textrm{d}x\textrm{d}t&= \tfrac{1}{2}\int _{\Omega }\big |\lambda g_o+(1-\lambda )v(0)-g_o\big |^2 \,\textrm{d}x\nonumber \\&= \tfrac{1}{2}(1-\lambda )^2\int _{\Omega } |v(0)-g_o|^2 \,\textrm{d}x. \end{aligned}$$

It remains to consider the second last term on the right-hand side of  (4.15), i.e. the integral

$$ -\tfrac{1}{2h_\ell }\iint _{\Omega \times [\tau -h_\ell , \tau ]} \big | v^{(h_\ell )} - u^{(h_\ell )} \big |^2\,\textrm{d}x\textrm{d}t. $$

Since $\partial _t g\in L^2(\Omega _T)$, we have

$$\begin{aligned} \lim _{\ell \rightarrow \infty } \tfrac{1}{2h_\ell } \iint _{\Omega \times [\tau -h_\ell ,\tau ]}|g^{(h_\ell )}-g|^2 \,\textrm{d}x\textrm{d}t\le \lim _{\ell \rightarrow \infty } \tfrac{1}{2} \iint _{\Omega \times [\tau -h_\ell ,\tau ]}|\partial _t g|^2 \,\textrm{d}x\textrm{d}t=0. \end{aligned}$$

Moreover, since *g* and *v* are of class $C^0([0,T];L^2(\Omega ))$, it follows that

$$\begin{aligned} \lim _{\ell \rightarrow \infty } \tfrac{1}{2h_\ell } \iint _{\Omega \times [\tau -h_\ell ,\tau ]}|g-g(\tau )|^2 +|v-v(\tau )|^2 \,\textrm{d}x\textrm{d}t=0. \end{aligned}$$

Finally, in view of (4.4) and the construction of $\widetilde{u}^{(h_\ell )}$, we find that

$$\begin{aligned} \lim _{\ell \rightarrow \infty }&\tfrac{1}{2h_\ell } \iint _{\Omega \times [\tau -h_\ell ,\tau ]}|u^{(h_\ell )}-\widetilde{u}^{(h_\ell )}(\tau )|^2 \,\textrm{d}x\textrm{d}t\\&\le \lim _{\ell \rightarrow \infty } h_\ell \bigg [\int _{\Omega \times \{\tau -h_\ell \}}f(\nabla u^{(h_\ell )})\,\textrm{d}x+ \iint _{\Omega \times [\tau -2h_\ell ,\tau ]} \big [\tfrac{1}{2}|\partial _tg|^2 + \mathsf K |\partial _t\nabla g|\big ]\,\textrm{d}x\textrm{d}t\bigg ] \\&\le \lim _{\ell \rightarrow \infty } h_\ell \bigg [|\Omega |\sup _{|\xi |\le L}|\nabla f(\xi )| + \iint _{\Omega _T} \big [\tfrac{1}{2}|\partial _tg|^2 +\mathsf K |\partial _t\nabla g|\big ]\,\textrm{d}x\textrm{d}t\bigg ] =0. \end{aligned}$$

This allows us to replace *g* and *v* by their restriction to the time slice $\Omega \times \{ \tau \}$, i.e. by $g(\tau )$ and $v(\tau )$, and $u^{(h_\ell )}$ by $u^{(h_\ell )}(\tau )$. It therefore remains to treat the integral

$$ \int _{\Omega }\big |\lambda g(\tau )+(1-\lambda )v(\tau )-\widetilde{u}^{(h_\ell )}(\tau )\big |^2 \,\textrm{d}x$$

in the limit $h_\ell \downarrow 0$. For this aim we need to identify the limit of $\widetilde{u}^{(h_\ell )}(\tau )$. We claim that $\widetilde{u}^{(h_\ell )}(\tau )\rightharpoondown u(\tau )$ weakly in $L^2(\Omega )$. Indeed, observing that $\widetilde{u}^{(h_\ell )}(0)=g_o$, we conclude for any $\eta \in L^2(\Omega )$ that

$$\begin{aligned} \lim _{\ell \rightarrow \infty } \int _{\Omega }\widetilde{u}^{(h_\ell )}(\tau )\eta \,\textrm{d}x&= \lim _{\ell \rightarrow \infty } \iint _{\Omega _\tau }\partial _t \widetilde{u}^{(h_\ell )}\eta \,\textrm{d}x\textrm{d}t+ \int _{\Omega }g_o\eta \,\textrm{d}x\\&= \iint _{\Omega _\tau }\partial _t u\,\eta \,\textrm{d}x\textrm{d}t+ \int _{\Omega }g_o\eta \,\textrm{d}x\\&= \int _{\Omega }u(\tau )\eta \, \textrm{d}x. \end{aligned}$$

By lower semicontinuity, we therefore have

4.19 $$\begin{aligned} \int _{\Omega }\big |\lambda g(\tau )&+(1-\lambda )v(\tau )-u(\tau )\big |^2 \textrm{d}x\nonumber \\&\le \liminf _{\ell \rightarrow \infty } \int _{\Omega }\big |\lambda g(\tau )+(1-\lambda )v(\tau )-\widetilde{u}^{(h_\ell )}(\tau )\big |^2 \textrm{d}x. \end{aligned}$$

Now we put everything together, i.e. we first use (4.16), (4.17), (4.18), and (4.19) in (4.15) to control the terms on the right-hand side, and then let $\ell \rightarrow \infty$. In this last argument, we use the fact that the integral functional related to the convex function *f* is lower semicontinuous w.r.t. the convergence $\nabla u^{(h_\ell )}\rightharpoondown \nabla u$ weakly in $L^{q}(\Omega _{T},\mathbb {R}^n)$ for any $q\ge 1$. With these arguments we obtain

$$\begin{aligned} \iint _{\Omega _\tau }&f(\nabla u)\,\textrm{d}x\textrm{d}t\\&\le (1-\lambda )\bigg [ \iint _{\Omega _\tau } f(\nabla v) \,\textrm{d}x\textrm{d}t+ \iint _{\Omega _\tau } \partial _t v(v-u) \,\textrm{d}x\textrm{d}t\bigg ] \\&\phantom {\le \ } +\lambda \bigg [ \iint _{\Omega _\tau } f(\nabla g) \,\textrm{d}x\textrm{d}t+ \iint _{\Omega _\tau } \partial _t g(v-u) \,\textrm{d}x\textrm{d}t\bigg ] \\&\phantom {=\ }- \tfrac{1}{2} \big \Vert (\lambda g +(1-\lambda )v-u)(\tau )\big \Vert ^2_{L^2(\Omega )} + \tfrac{1}{2}(1-\lambda )^2 \Vert v(0)-g_o\Vert ^2_{L^2(\Omega )} . \end{aligned}$$

Letting $\lambda \downarrow 0$ gives that

$$\begin{aligned} \iint _{\Omega _\tau } f(\nabla u)\,\textrm{d}x\textrm{d}t&\le \iint _{\Omega _\tau } f(\nabla v) \,\textrm{d}x\textrm{d}t+ \iint _{\Omega _\tau } \partial _t v(v-u) \,\textrm{d}x\textrm{d}t\\&\phantom {=\ }- \tfrac{1}{2} \Vert (v-u)(\tau )\Vert ^2_{L^2(\Omega )} + \tfrac{1}{2}\Vert v(0)-g_o\Vert ^2_{L^2(\Omega )} . \end{aligned}$$

This inequality applies to all $v\in g+ L^\infty (0,T;W^{1,\infty }_{0}(\Omega ))$ satisfying the requirements

$$ \Vert \nabla v\Vert _{L^\infty (\Omega _T ,\mathbb {R}^n)} \le L,\quad \partial _t v\in L^2(\Omega _T), \quad \text{ and } v(0)\in L^2(\Omega ). $$

Hence, *u* is a variational solution of the gradient constrained problem. The uniqueness can be deduced as in [9, Lemma 3.3]. This finishes the proof of Proposition 4.1.

## Existence of Solutions to the Gradient Constrained Problem

In this section we prove the existence of a variational solution to the gradient constrained problem in the sense of Definition 1.2 under the assumptions of Theorem 1.3. In particular, we assume the boundary data *g* to admit merely the regularity given in hypothesis (1.2).

### Proposition 5.1

Let $f:\mathbb {R}^n\rightarrow \mathbb {R}$ be convex, $L>0$ and suppose that *g* satisfies (1.2) and (1.5). Then, there exists a unique variational solution *u* in $\Omega _T$ in the class $\textrm{Lip}^x_g(\Omega _T,L)$ in the sense of Definition 1.2.

For the proof of Proposition 5.1 we will rely on the existence result from Proposition 4.1 for more regular boundary data. Therefore, we first regularize the boundary data in such a way that Proposition 4.1 is applicable. Subsequently we will show that the limit function is a solution to the original problem.

### Construction of Regularized Data

We define *regularized boundary values* $g_i$, $i\in \mathbb {N}$, according to (2.2) with $(g_o, g,h_i)$ instead of $(v_o, v,h)$. Recall that

5.1 $$\begin{aligned} g_i(t):= [g]_{h_i}(t)= \textrm{e}^{-\frac{t}{h_i}}g_{o}+ \tfrac{1}{h_i}\int _0^t\textrm{e}^\frac{s-t}{h_i}g(s)\,\textrm{d}s. \end{aligned}$$

Since $g\in L^q\big ( 0,T;W^{1,q}(\Omega )\big )$ and $g_o\in W^{1,q}(\Omega )$ for any $q\ge 1$, we may use the assertions of Lemma 2.6 with $r=q$ and $X=W^{1,q}(\Omega )$. From Lemma 2.6 we get $g_i\in L^{q}(0,T;W^{1,q}(\Omega ))$ with $\partial _{t}g_i\in L^{2}(\Omega _{T})$. Moreover, we have

5.2 $$\begin{aligned} \partial _{t}g_i=\tfrac{1}{h_i}(g-g_i)\in L^2(\Omega _T) \text{ and } \partial _t\nabla g_i=\tfrac{1}{h_i}(\nabla g-\nabla g_i)\in L^q(\Omega _T,\mathbb {R}^n). \end{aligned}$$

The second assertion follows, as the right-hand side of the identity for $\partial _{t}g_i$ belongs to $L^{q}\big (0,T;W^{1,q}(\Omega )\big )$. Since $g_i(0)=g_{o}$, we also have $\partial _{t}g_i(0)=\frac{1}{h_i}(g_{o}-g_i(0))=0$. From the second part of Lemma 2.6 we obtain

5.3 $$\begin{aligned} \Vert \partial _t g_i\Vert _{L^2(\Omega _{t_o})} \le \Vert \partial _t g\Vert _{L^2(\Omega _{t_o})} \quad \text{ for } \text{ any } t_o\in (0,T]. \end{aligned}$$

Further, using the convexity of $\xi \mapsto |\xi |^q$ and Jensen’s inequality we conclude from (5.1) that

$$\begin{aligned} |\nabla g_i(t)|^q&= \bigg | \textrm{e}^{-\frac{t}{h_i}}\nabla g_{o} +\frac{1-\textrm{e}^{-\frac{t}{h_i}}}{h_i(1-\textrm{e}^{-\frac{t}{h_i}})} \int _0^t\textrm{e}^\frac{s-t}{h_i}\nabla g(s)\,\textrm{d}s\bigg |^q\\&\le \textrm{e}^{-\frac{t}{h_i}}|\nabla g_o|^q + \big ( 1-\textrm{e}^{-\frac{t}{h_i}}\big ) \bigg |\tfrac{1}{h_i(1-\textrm{e}^{-\frac{t}{h_i}})} \int _0^t\textrm{e}^\frac{s-t}{h_i}\nabla g(s)\,\textrm{d}s\bigg |^q\\&\le \textrm{e}^{-\frac{t}{h_i}}|\nabla g_o|^q + \tfrac{1}{h_i} \int _0^t\textrm{e}^\frac{s-t}{h_i}|\nabla g(s)|^q\,\textrm{d}s\\&\le \bigg [ \textrm{e}^{-\frac{t}{h_i}}\Vert \nabla g_o\Vert ^q_{L^\infty (\Omega ,\mathbb {R}^n)} + \tfrac{1}{h_i}\int _0^t\textrm{e}^\frac{s-t}{h_i}\Vert \nabla g(s)\Vert ^q_{L^\infty (\Omega ,\mathbb {R}^n)}\,\textrm{d}s\bigg ]\\&\le \bigg [ \textrm{e}^{-\frac{t}{h_i}}\Vert \nabla g_o\Vert _{L^\infty (\Omega ,\mathbb {R}^n)}^q +\big (1 -\textrm{e}^{-\frac{t}{h_i}}\big ) \Vert \nabla g\Vert _{L^\infty (\Omega _T,\mathbb {R}^n)}^q\bigg ]\\&\le \max \Big \{ \Vert \nabla g_o\Vert _{L^\infty (\Omega ,\mathbb {R}^n)}^q, \Vert \nabla g\Vert _{L^\infty (\Omega _T,\mathbb {R}^n)}^q\Big \}. \end{aligned}$$

Therefore we haveThis, however, implies the $L^\infty$-gradient bound

5.4 $$\begin{aligned} \Vert \nabla g_i\Vert _{L^\infty (\Omega _T,\mathbb {R}^n)} \le \max \big \{ \Vert \nabla g_o\Vert _{L^\infty (\Omega ,\mathbb {R}^n)}, \Vert \nabla g\Vert _{L^\infty (\Omega _T,\mathbb {R}^n)} \big \}<L. \end{aligned}$$

Moreover, from (5.2) and (5.4) we conclude

5.5 $$\begin{aligned} \partial _t g_i\in L^\infty \big ( 0,T; W^{1,\infty }(\Omega )\big ). \end{aligned}$$

The task now is to construct suitable comparison functions that match the regularized boundary values $g_i$ on the lateral boundary. The difficulty here is that these must be constructed from a generic comparison function *v* that coincides with *g* on the lateral boundary of $\Omega _T$. Recall that a general comparison function $v\in g+L^\infty \big (0,T; W^{1,\infty }_0(\Omega )\big )$ satisfies $\partial _tv\in L^2(\Omega _T)$, $v(0)\in L^2(\Omega )$, and

$$ \Vert \nabla v\Vert _{L^\infty (\Omega _T,\mathbb {R}^n)}\le L. $$

We construct the comparison maps $v_i$ using the same procedure that we used to construct the regularized boundary values $g_i$, i.e. we define $v_i:=[v]_{h_i}$. Note that the time mollification in (2.2) is performed with $g_o$ instead of *v*(0) as the initial value, i.e.

$$\begin{aligned} v_i(t):= [v]_{h_i}(t)= \textrm{e}^{-\frac{t}{h_i}}g_{o}+\tfrac{1}{h_i}\int _0^t\textrm{e}^\frac{s-t}{h_i}v(s)\,\textrm{d}s\quad \text{ for } \text{ any } i\in \mathbb {N}. \end{aligned}$$

The question naturally arises why $g_o$ and not *v*(0) is used as the initial value. This is because the gradient constraint must be satisfied for the time mollification $v_i$. This, however, is not possible with the initial values *v*(0), since $v(0)\in L^2(\Omega )$ but not necessarily in $W^{1,\infty }(\Omega )$. Lemma 2.6 ensures that $\partial _t v_i\in L^2(\Omega _T)$. Since $v\in L^q\big ( 0,T; W^{1,q}(\Omega )\big )$ and $g_o\in W^{1,q}(\Omega )$ for any $q\ge 1$, Lemma 2.6 with $r=q$ and $X=W^{1,q}(\Omega )$ yields $v_i\in L^{q}(0,T;W^{1,q}(\Omega ))$, and as for the $g_i$ we obtainso that

$$ \Vert \nabla v_i\Vert _{L^\infty (\Omega _T,\mathbb {R}^n)} \le \max \big \{ \Vert \nabla g_o\Vert _{L^\infty (\Omega ,\mathbb {R}^n)}, \Vert \nabla v\Vert _{L^\infty (\Omega _T,\mathbb {R}^n)} \big \}\le L. $$

Hence, $v_i\in g_i+L^\infty \big ( 0,T; W^{1,\infty }_0(\Omega )\big )$. Furthermore, by Lemma 2.6 we have

5.6 $$\begin{aligned} v_i\rightarrow v \text{ in } L^q(\Omega _T) \text{ in } \text{ the } \text{ limit } i\rightarrow \infty \end{aligned}$$

for any $$q\ge 1$$.

Next we prove that the sequence $(\partial _t v_i)_{i\in \mathbb {N}}$ is uniformly bounded in $L^1\big ( 0,T;L^2(\Omega )\big )$. For this purpose we define that

5.7 $$\begin{aligned} \widetilde{v}_i(t) := v_i(t)+\mathrm e^{-\frac{t}{h_i}}(v(0)-g_o) = \textrm{e}^{-\frac{t}{h_i}}v(0)+\tfrac{1}{h_i} \int _0^t\textrm{e}^\frac{s-t}{h_i}v(s)\,\textrm{d}s, \end{aligned}$$

so that $\widetilde{v}_i(0)=v(0)$. Correcting the initial values of $v_i$ from $g_o$ to *v*(0) allows the application of the part of Lemma 2.6 (with $r=2$, $X=L^2(\Omega )$) that refers to the time derivative of the mollification, because $\partial _tv\in L^2(\Omega _T)=L^2\big ( 0,T, L^2(\Omega )\big )$ by assumption. By Lemma 2.6 we obtain

$$\begin{aligned} \int _0^T\big \Vert \partial _t\widetilde{v}_i(t)\big \Vert _{L^2(\Omega )}\textrm{d}t&\le \sqrt{T}\big \Vert \partial _t\widetilde{v}_i\big \Vert _{L^2(\Omega _T)} \le \sqrt{T}\Vert \partial _tv\Vert _{L^2(\Omega _T)}. \end{aligned}$$

Moreover, we have

$$\begin{aligned} \int _0^T \big \Vert \partial _t\big ( \mathrm e^{-\frac{t}{h_i}}(v(0)-g_o)\big )\big \Vert _{L^2(\Omega )}\textrm{d}t&=\int _0^T\tfrac{1}{h_i}\mathrm e^{-\frac{t}{h_i}}\textrm{d}t\, \Vert v(0)-g_o\Vert _{L^2(\Omega )}\\&\le \Vert v(0)-g_o\Vert _{L^2(\Omega )}. \end{aligned}$$

This is exactly the reason why the $L^1\big (0,T; L^2(\Omega )\big )$-norm comes into play, because for any other exponents $r>1$ we only have

$$\begin{aligned} \int _0^T \big \Vert \partial _t\big ( \mathrm e^{-\frac{t}{h_i}}(v(0)-g_o)\big )\big \Vert _{L^2(\Omega )}^r\textrm{d}t&=\int _0^T\tfrac{1}{h_i^r}\mathrm e^{-\frac{rt}{h_i}}\textrm{d}t\, \Vert v(0)-g_o\Vert _{L^2(\Omega )}^r\\&= \frac{1-\mathrm e^{-\frac{rT}{h_i}}}{rh_i^{r-1}}\, \Vert v(0)-g_o\Vert _{L^2(\Omega )}^r\rightarrow \infty \end{aligned}$$

in the limit $i\rightarrow \infty$, which means that there is no uniform $L^r\big (0,T; L^2(\Omega )\big )$-bound with $r>1$ for $\mathrm e^{-\frac{t}{h_i}}(v(0)-g_o)$. Together, the second and third last inequalities and (5.7) imply

5.8 $$\begin{aligned} \int _0^T\Vert \partial _t v_i(t)\Vert _{L^2(\Omega )}\textrm{d}t&\le \sqrt{T}\Vert \partial _tv\Vert _{L^2(\Omega _T)}+\Vert v(0)-g_o\Vert _{L^2(\Omega )}. \end{aligned}$$

Finally, Lemma 2.6 applied with $r=q$ and $X=L^q(\Omega ,\mathbb {R}^n)$ yields that

5.9 $$\begin{aligned} \nabla v_i \rightarrow \nabla v \text{ in } L^q(\Omega _T,\mathbb {R}^n) \text{ as } i\rightarrow \infty \end{aligned}$$

for any $q\ge 1$. We note that (5.8) and (5.9) apply in particular for the choice $v=g$, since *g* is admissible as comparison map.

### The Regularized Problem Formulation

According to (5.4) and (5.5), the regularized boundary values $g_i$ defined in (5.1) satisfy all the conditions assumed in Chapter 4. Hence the application of Proposition 4.1 ensures the existence of variational solutions $u_i\in \textrm{Lip}^x_{g_i}(\Omega _T,L)$ to the variational inequalities

5.10 $$\begin{aligned} \iint _{\Omega _\tau }f(\nabla u_i)\,\textrm{d}x\textrm{d}t&\le \iint _{\Omega _\tau }\partial _tv(v-u_i)\,\textrm{d}x\textrm{d}t+ \iint _{\Omega _\tau }f(\nabla v)\,\textrm{d}x\textrm{d}t\nonumber \\&\phantom {\le \,} -\tfrac{1}{2}\Vert (v-u_i)(\tau )\Vert ^2_{L^2(\Omega )}+\tfrac{1}{2}\Vert v(0)-g_o\Vert ^2_{L^2(\Omega )}. \end{aligned}$$

These hold true for any $\tau \in [0,T]$ and any $v\in \textrm{Lip}^x_{g_i}(\Omega _T,L)$ with $\partial _tv\in L^2(\Omega _T)$ and $v(0)\in L^2(\Omega )$. We let $t_o\in (0,T]$ and consider $\tau \in [0,t_o]$ in (5.10). Choosing the testing function $v=g_i$ (recall that $g_i$ is admissible) and discarding the positive term on the right-hand side, we obtain for any $\tau \in [0,t_o]$ that

$$\begin{aligned}&\tfrac{1}{2}\Vert (g_i-u_i)(\tau )\Vert ^2_{L^2(\Omega )}\\&\quad \le \iint _{\Omega _\tau }|\partial _t g_i| |g_i-u_i|\,\textrm{d}x\textrm{d}t+ |\Omega _\tau |\sup _{|\xi |\le L} f(\xi ) \\&\quad \le \tfrac{1}{4} \sup _{t\in [0,t_o]} \Vert (g_i-u_i)(t)\Vert ^2_{L^2(\Omega )} + t_o\Vert \partial _tg_i\Vert ^2_{L^2(\Omega _{t_o})} + |\Omega _{t_o}|\sup _{|\xi |\le L} f(\xi ). \end{aligned}$$

Taking the supremum with respect to $\tau \in [0,t_o]$, we may re-absorb the first term on the right into the left. Using the bound (5.3) for $\partial _tg_i$ to estimate the second term on the right, we obtain that for any $t_o\in (0,T]$ that

5.11 $$\begin{aligned} \sup _{t\in [0,t_o]} \Vert (g_i-u_i)(t)\Vert ^2_{L^2(\Omega )} \le 4t_o\Vert \partial _tg\Vert ^2_{L^2(\Omega _{t_o})} + 4|\Omega _{t_o}|\sup _{|\xi |\le L} f(\xi ). \end{aligned}$$

In particular for the choice $t_o=T$, we obtain an $L^\infty {-}L^2$ estimate for the solutions $u_i$. Indeed, we have

$$\begin{aligned} \sup _{t\in [0,T]}\Vert u_i(t)\Vert ^2_{L^2(\Omega )} \le 2 \sup _{t\in [0,T]}\Vert g_i(t)\Vert ^2_{L^2(\Omega )} + 8T \Vert \partial _tg\Vert ^2_{L^2(\Omega _T)} + 8|\Omega _T| \sup _{|\xi |\le L} f(\xi ). \end{aligned}$$

The first term on the right-hand side is uniformly (with respect to $i\in \mathbb {N}$) bounded with respect to $i\in \mathbb {N}$ according to Lemma 2.6 applied with $r=\infty$ and $X=L^2(\Omega )$. Therefore, the above estimate together with the uniform gradient bound $\Vert \nabla u_i\Vert _{L^\infty (\Omega _T,\mathbb {R}^n)}\le L$ imply that the sequence $(u_i)_{i\in \mathbb {N}}$ is uniformly bounded in $L^\infty \big ( 0,T;L^2(\Omega )\big )$ and $L^\infty \big ( 0,T; W^{1,\infty }(\Omega )\big )$.

### Limit Passage and Convergence

Due to the uniform estimates there exists a function

$$ u\in L^{\infty }\big (0,T; L^2(\Omega )\big )\cap \bigcap _{q\ge 1}L^{q}\big (0,T;W^{1,p}(\Omega )\big ) $$

and a subsequence of $u_{i}$ (still denoted this way) such that

5.12 $$\begin{aligned} \left\{ \begin{array}{cl} u_{i}\rightharpoonup u &  \text{ weakly } \text{ in } L^{q}\big (0,T; W^{1,q}(\Omega )\big ) \text{ for } \text{ any } q\ge 1,\\ u_{i}\rightharpoonup u &  \text{ weakly}^* \text{ in } L^{\infty }\big (0,T, L^2(\Omega )\big ), \end{array}\right. \end{aligned}$$

as $i\rightarrow \infty$. At this point we need to show that *u* is the sought variational solution with lateral boundary values *g* and initial value $g_o$, that satisfies the gradient constraint. The latter results from the weak $L^p$-convergence of the gradients and the lower-semicontinuity of the $L^q$-norm with respect to weak convergence. More precisely, for any $q\ge 1$ we haveso that the gradient constraint $\Vert \nabla u\Vert _{L^\infty (\Omega _T,\mathbb {R}^n)}\le L$ holds. In order to check that *u* solves the variational inequality we consider an arbitrary comparison function $v\in \textrm{Lip}^x_g(\Omega _T,L)$ with $\partial _tv\in L^2(\Omega )$ and $v(0)\in L^2(\Omega )$. As before, we write $v_i:=[v]_{h_i}$ for the time mollification of *v* and with initial value $g_o$. As shown in § 5, the $v_i$ are admissible in the variational inequality (5.10), from which we obtain

5.13 $$\begin{aligned} \tfrac{1}{2} \Vert (v_i-u_i)(\tau )\Vert ^2_{L^2(\Omega )}&+\iint _{\Omega _\tau } f(\nabla u_i)\,\textrm{d}x\textrm{d}t\nonumber \\&\le \iint _{\Omega _\tau }\partial _tv_i(v_i-u_i)\,\textrm{d}x\textrm{d}t+ \iint _{\Omega _\tau } f(\nabla v_i)\,\textrm{d}x\textrm{d}t\end{aligned}$$

for any $\tau \in [0,T]$. Note that $v_i$ and $g_i$ attend the same initial datum $g_o$, so that the $L^2$-boundary term vanishes for $t=0$. Now, the overall goal is to pass in (5.13) to the limit $i\rightarrow \infty$. Hereby, the degree of difficulty in the treatment of the individual terms is quite different. We start with the second integral on the right. Since both functions *v* and $v_i$ satisfy the gradient constraint we have

5.14 $$\begin{aligned} \bigg |\iint _{\Omega _\tau } f(\nabla v_i)\,\textrm{d}x\textrm{d}t&-\iint _{\Omega _\tau } f(\nabla v)\,\textrm{d}x\textrm{d}t\bigg |\nonumber \\&\le \sup _{|\xi |\le L} |\nabla f(\xi )|\iint _{\Omega _T} | \nabla v_i-\nabla v|\,\textrm{d}x\textrm{d}t. \end{aligned}$$

Because of (5.9) with $q=1$, the integral on the right converges to 0. This allows us to replace the second integral on the right-hand side of (5.13) by $\iint _{\Omega _\tau } f(\nabla v)\,\textrm{d}x\textrm{d}t$ after passing to the limit $i\rightarrow \infty$. In the first integral, i.e. the integral containing the time derivative, we replace $\partial _t v_i$ by $\partial _t\widetilde{v}_i$, where $\widetilde{v}_i$ is defined in (5.7). We recall that passing from $v_i$ to $\widetilde{v}_i$ means just a correction of the initial values. We have

5.15 $$\begin{aligned} \iint _{\Omega _\tau }&\partial _tv_i(v_i-u_i)\,\textrm{d}x\textrm{d}t\nonumber \\&= \iint _{\Omega _\tau }\partial _t\widetilde{v}_i(v_i-u_i)\,\textrm{d}x\textrm{d}t+ \tfrac{1}{h_i} \iint _{\Omega _\tau }\mathrm e^{-\frac{t}{h_i}}(v(0)-g_o)(v_i-u_i)\,\textrm{d}x\textrm{d}t. \end{aligned}$$

In view of the second part of Lemma 2.6 we have $\partial _t\widetilde{v}_i(t) = \frac{1}{h_i}\int _0^t\mathrm e^\frac{s-t}{h_i}\partial _sv(s)\,\textrm{d}s$. Thus the first part of Lemma 2.6 can be applied to $\partial _tv\in L^2(\Omega _T)=L^2\big ( 0,T;L^2(\Omega )\big )$ with the choice $v_o=0$. This yields $\partial _t\widetilde{v}_i\rightarrow \partial _tv$ in $L^2(\Omega _T)$ as $i\rightarrow \infty$. Combining this observation with (5.6) and (5.12)$_2$, we get for the first term on the left

5.16 $$\begin{aligned} \lim _{i\rightarrow \infty } \iint _{\Omega _\tau }\partial _t\widetilde{v}_i(v_i-u_i)\,\textrm{d}x\textrm{d}t= \iint _{\Omega _\tau }\partial _t v(v-u)\,\textrm{d}x\textrm{d}t. \end{aligned}$$

Next, we write the second integral on the right-hand side of (5.15) in the form

$$\begin{aligned} \tfrac{1}{h_i} \iint _{\Omega _\tau }\mathrm e^{-\frac{t}{h_i}}(v(0)-g_o)(v_i-u_i)\,\textrm{d}x\textrm{d}t&= \textbf{I}_i+\textbf{II}_i+\textbf{III}_i+\textbf{IV}_i, \end{aligned}$$

where we abbreviated

$$\begin{aligned} \textbf{I}_i&:=\tfrac{1}{h_i}\iint _{\Omega _\tau }\Big (\mathrm e^{-\frac{2t}{h_i}}- \mathrm e^{-\frac{t}{h_i}}\Big ) (v(0)-g_o)g_o\,\textrm{d}x\textrm{d}t,\\ \textbf{II}_i&:=\tfrac{1}{h_i^2}\iint _{\Omega _\tau }\bigg [\mathrm e^{-\frac{2t}{h_i}} (v(0)-g_o)\int _0^t \mathrm e^\frac{s}{h_i}v(0)\textrm{d}s\bigg ]\,\textrm{d}x\textrm{d}t,\\ \textbf{III}_i&:=\tfrac{1}{h_i^2}\iint _{\Omega _\tau }\bigg [\mathrm e^{-\frac{2t}{h_i}} (v(0)-g_o)\int _0^t \mathrm e^\frac{s}{h_i}(v(s)-v(0))\textrm{d}s\bigg ]\,\textrm{d}x\textrm{d}t,\\ \textbf{IV}_i&:=\tfrac{1}{h_i}\iint _{\Omega _\tau } \mathrm e^{-\frac{t}{h_i}} (v(0)-g_o)(g_o-u_i)\,\textrm{d}x\textrm{d}t. \end{aligned}$$

In $\textbf{I}_i$ we first compute the one-dimesional integral with respect to *t* and then pass to the limit $i\rightarrow \infty$. This leads to

$$\begin{aligned} \lim _{i\rightarrow \infty }\textbf{I}_i&= \lim _{i\rightarrow \infty } \tfrac{1}{h_i}\int _0^\tau \Big (\mathrm e^{-\frac{2t}{h_i}}- \mathrm e^{-\frac{t}{h_i}}\Big ) \,\textrm{d}t\int _\Omega (v(0)-g_o)g_o\,\textrm{d}x\\&=-\tfrac{1}{2} \int _\Omega (v(0)-g_o)g_o\,\textrm{d}x. \end{aligned}$$

The second integral $\textbf{II}_i$ is treated analogously. We obtain

$$\begin{aligned} \lim _{i\rightarrow \infty }\textbf{II}_i&= \lim _{i\rightarrow \infty } \tfrac{1}{h_i^2}\int _0^\tau \mathrm e^{-\frac{2t}{h_i}} \int _0^t\mathrm e^{\frac{s}{h_i}}\,\textrm{d}s\textrm{d}t\int _\Omega (v(0)-g_o)v(0)\,\textrm{d}x\\&=\tfrac{1}{2} \int _\Omega (v(0)-g_o)v(0)\,\textrm{d}x. \end{aligned}$$

Together with the result for $\textbf{I}_i$ this gives

$$\begin{aligned} \lim _{i\rightarrow \infty }\big [ \textbf{I}_i+\textbf{II}_i\big ]&=\tfrac{1}{2} \Vert v(0)-g_o\Vert ^2_{L^2(\Omega )}, \end{aligned}$$

which is exactly the $L^2(\Omega )$-boundary term at $t=0$. Next, we treat the term $\textbf{III}_i$. This integral can can be estimated with Hölder’s inequality. We obtain

$$\begin{aligned} \lim _{i\rightarrow \infty }&\textbf{III}_i\\&\le \Vert v(0)-g_o\Vert _{L^2(\Omega )} \lim _{i\rightarrow \infty }\tfrac{1}{h_i^2}\int _0^\tau \mathrm e^{-\frac{2t}{h_i}} \int _0^t \mathrm e^\frac{s}{h_i} \bigg [ \int _\Omega |v(s)-v(0)|^2\,\textrm{d}x\bigg ]^\frac{1}{2}\,\textrm{d}s\textrm{d}t\\&= \Vert v(0)-g_o\Vert _{L^2(\Omega )} \lim _{i\rightarrow \infty }\tfrac{1}{h_i^2}\int _0^\tau \mathrm e^{-\frac{2t}{h_i}} \int _0^t \mathrm e^\frac{s}{h_i} \bigg [ \int _\Omega \bigg |\int _0^s\partial _\sigma v(\sigma )\textrm{d}\sigma \bigg |^2\,\textrm{d}x\bigg ]^\frac{1}{2}\,\textrm{d}s\textrm{d}t\\&\le \Vert v(0)-g_o\Vert _{L^2(\Omega )}\Vert \partial _tv\Vert _{L^2(\Omega _\tau )} \lim _{i\rightarrow \infty }\tfrac{1}{h_i^2}\int _0^\tau \mathrm e^{-\frac{2t}{h_i}}\int _0^t\sqrt{s}\mathrm e^\frac{s}{h_i}\,\textrm{d}s\textrm{d}t\\&\le \tfrac{2}{3} \Vert v(0)-g_o\Vert _{L^2(\Omega )}\Vert \partial _tv\Vert _{L^2(\Omega _\tau )} \lim _{i\rightarrow \infty }\tfrac{1}{h_i^2} \int _0^\tau \mathrm e^{-\frac{t}{h_i}}t^\frac{3}{2}\,\textrm{d}t\\&\le \tfrac{2}{3} \Vert v(0)-g_o\Vert _{L^2(\Omega )}\Vert \partial _tv\Vert _{L^2(\Omega _\tau )} \int _0^\infty s^\frac{3}{2}\mathrm e^{-s}\,\textrm{d}s\lim _{i\rightarrow \infty }\sqrt{h_i} = 0. \end{aligned}$$

Finally, we deal with term $\textbf{IV}_i$, which is the most difficult one. First, we apply the Cauchy-Schwarz inequality in space. In the resulting integral we decompose the time interval $[0,\tau ]$ into two parts, i.e. in $[0,\sqrt{h_i}]$ and $(\sqrt{h_i},\tau ]$. This leads to

$$\begin{aligned} | \textbf{IV}_i|&\le \Vert v(0)-g_o\Vert _{L^2(\Omega )} \tfrac{1}{h_i}\int _0^\tau \mathrm e^{-\frac{t}{h_i}}\Vert g_o-u_i(t)\Vert _{L^2(\Omega )}\,\textrm{d}t\\&= \Vert v(0)-g_o\Vert _{L^2(\Omega )} \big [ \textbf{IV}_i^{(1)} +\textbf{IV}_i^{(2)} \big ], \end{aligned}$$

where we used the shorthand notation

$$\begin{aligned} \textbf{IV}_i^{(1)}&:=\tfrac{1}{h_i}\int _0^{\sqrt{h_i}} \mathrm e^{-\frac{t}{h_i}}\Vert g_o-u_i(t)\Vert _{L^2(\Omega )}\,\textrm{d}t,\\ \textbf{IV}_i^{(2)}&:= \tfrac{1}{h_i}\int _{\sqrt{h_i}}^\tau \mathrm e^{-\frac{t}{h_i}}\Vert g_o-u_i(t)\Vert _{L^2(\Omega )}\,\textrm{d}t. \end{aligned}$$

Here the second term $\textbf{IV}_i^{(2)}$ is easier to deal with, and therefore we start with it. Using the uniform $L^\infty (0,T;L^2(\Omega ))$-bound for $u_i$ we obtain

$$\begin{aligned} \lim _{i\rightarrow \infty }\textbf{IV}_i^{(2)}&\le \lim _{i\rightarrow \infty }\Vert g_o-u_i\Vert _{L^\infty (0,T;L^2(\Omega ))} \tfrac{1}{h_i}\int _{\sqrt{h_i}}^\tau \mathrm e^{-\frac{t}{h_i}}\,\textrm{d}t\\&\le \sup _{i\in \mathbb {N}} \Vert g_o-u_i\Vert _{L^\infty (0,T;L^2(\Omega ))} \lim _{i\rightarrow \infty } \Big [ \mathrm e^{-\frac{1}{\sqrt{h_i}}}-\mathrm e^{-\frac{\tau }{h_i}}\Big ]\\&\le \sup _{i\in \mathbb {N}} \Vert g_o-u_i\Vert _{L^\infty (0,T;L^2(\Omega ))} \lim _{i\rightarrow \infty } \mathrm e^{-\frac{1}{\sqrt{h_i}}} =0. \end{aligned}$$

Thus, it remains to consider the term $\textbf{IV}_i^{(1)}$. We have

$$\begin{aligned} \textbf{IV}_i^{(1)}&\le \Vert g_o-u_i\Vert _{L^\infty (0,\sqrt{h_i};L^2(\Omega ))} \tfrac{1}{h_i}\int _0^{\sqrt{h_i}} \mathrm e^{-\frac{t}{h_i}}\,\textrm{d}t\\&= \Vert g_o-u_i\Vert _{L^\infty (0,\sqrt{h_i};L^2(\Omega ))} \Big ( 1-\mathrm e^{-\frac{1}{\sqrt{h_i}}}\Big )\\&\le \Vert g_o-u_i\Vert _{L^\infty (0,\sqrt{h_i};L^2(\Omega ))}. \end{aligned}$$

From (5.11) applied with $t_o=\sqrt{h_i}$ we obtain

$$\begin{aligned} \lim _{i\rightarrow \infty } \sup _{t\in [0,\sqrt{h_i}]} \Vert (g_i-u_i)(t)\Vert ^2_{L^2(\Omega )} \le 4\lim _{i\rightarrow \infty } \sqrt{h_i} \Big [\Vert \partial _tg\Vert ^2_{L^2(\Omega _{T})}{ +} |\Omega |\sup _{|\xi |\le L} f(\xi )\Big ] =0. \end{aligned}$$

At this point, it remains to analyze the difference between $g_i(t)$ and $g_o$ close to $t=0$. By (5.1) we have for any $t\in [0,\sqrt{h_i}]$ that

$$\begin{aligned} \Vert g_i(t)-g_o\Vert ^2_{L^2(\Omega )}&= \int _{\Omega }\bigg |\big (\mathrm e^{-\frac{t}{h_i}}-1\big ) g_o + \tfrac{1}{h_i}\int _0^t\textrm{e}^\frac{s-t}{h_i}g(s)\,\textrm{d}s\bigg |^2 \,\textrm{d}x\\&= \int _{\Omega }\bigg | \tfrac{1}{h_i}\int _0^t\textrm{e}^\frac{s-t}{h_i}(g(s)-g_o)\,\textrm{d}s\bigg |^2 \,\textrm{d}x\\&\le \sup _{\tau \in [0,\sqrt{h_i}]} \Vert g(\tau )-g_o \Vert ^2_{L^2(\Omega )}. \end{aligned}$$

Taking the supremum with respect to *t* over the interval $[0,\sqrt{h_i}]$, we find

$$\begin{aligned} \sup _{t\in [0,\sqrt{h_i}]} \Vert g_i(t)-g_o\Vert ^2_{L^2(\Omega )}&\le \sup _{t\in [0,\sqrt{h_i}]} \Vert g(t)-g_o \Vert ^2_{L^2(\Omega )}. \end{aligned}$$

Since $g\in C^0\big ( [0,T]; L^2(\Omega )\big )$ with $g(0)=g_o$, the right-hand side of the preceding inequality converges to 0 as $i\rightarrow \infty$. The combination of the last inequalities implies that also $\textbf{IV}_i^{(1)}\rightarrow 0$ in the limit $i\rightarrow \infty$. Therefore we have everything at hand to pass to the limit in (5.15). For a.e. $\tau \in (0,T)$ we get

5.17 $$\begin{aligned} \lim _{i\rightarrow \infty } \iint _{\Omega _\tau }&\partial _tv_i(v_i-u_i)\,\textrm{d}x\textrm{d}t= \iint _{\Omega _\tau }\partial _t v(v-u)\,\textrm{d}x\textrm{d}t+ \tfrac{1}{2} \Vert v(0)-g_o\Vert ^2_{L^2(\Omega )}. \end{aligned}$$

Moreover, the first integral on the right-hand side can be uniformly bounded with respect to $\tau \in (0,T)$ and $i\in \mathbb {N}$ using (5.8). Indeed, we have

5.18 $$\begin{aligned} \sup _{i\in \mathbb {N}}&\sup _{\tau \in (0,T)}\bigg |\iint _{\Omega _\tau }\partial _tv_i(v_i-u_i)\,\textrm{d}x\textrm{d}t\bigg |\nonumber \\&\le \sup _{i\in \mathbb {N}}\Vert \partial _tv_i\Vert _{L^1(0,T;L^2(\Omega ))}\Vert v_i-u_i\Vert _{L^\infty (0,T;L^2(\Omega ))}\nonumber \\&\le \sqrt{T}\Vert \partial _tv\Vert _{L^2(\Omega _T)}\sup _{i\in \mathbb {N}}\Vert v_i-u_i\Vert _{L^\infty (0,T;L^2(\Omega ))}<\infty . \end{aligned}$$

Before proceeding to the limit $i\rightarrow \infty$ in (5.13), we integrate both sides with respect to $\tau$ over time intervals $(t_o, t_o+\delta )$ contained in (0, *T*) and then take means on both sides. In this way we get5.19The integrals on the left-hand side are both lower semicontinuous. Note that according to (5.6) $v_i$ converges strongly in $L^2(\Omega )$ to *v*, that *f* is convex, and that according to (5.12)$_1$ $\nabla u_i$ converges weakly in $L^q(\Omega _T,\mathbb {R}^n)$ to $\nabla u$ for any $q\ge 1$. According to (5.14) the integral of $f(\nabla v_i)$ converges to the corresponding integral of $f(\nabla v)$. Finally, by the dominated convergence theorem we can also pass to the limit $i\rightarrow \infty$ in the integral on the right-hand side containing the time derivative. Recall that for a.e. $\tau$ the functions $\tau \mapsto \iint _{\Omega _\tau }\partial _tv_i(v_i-u_i)\,\textrm{d}x\textrm{d}t$ are converging, i.e. on the one hand we have a.e. convergence on (0, *T*), and on the other hand, the functions are uniformly bounded with respect to *i* and $\tau$. In this way we getfor any $t_o\in [0,T]$ and any $\delta \in (0,T-t_o]$. Here we let $\delta \downarrow 0$ and obtain

$$\begin{aligned} \tfrac{1}{2} \Vert (v&-u)(t_o)\Vert ^2_{L^2(\Omega )} + \iint _{\Omega _{t_o}} f(\nabla u)\,\textrm{d}x\textrm{d}t\nonumber \\&\le \iint _{\Omega _{t_o}}\big [\partial _tv(v-u)+f(\nabla v)\big ]\textrm{d}x\textrm{d}t+\tfrac{1}{2} \Vert v(0)-g_o\Vert ^2_{L^2(\Omega )} \end{aligned}$$

for a.e. $t_o\in [0,T]$. Overall, we have shown that *u* is a variational solution of the gradient constraint problem. The uniqueness can be deduced as in [9, Lemma 3.3]. This finishes the proof of Proposition 5.1.

## Eliminating the Gradient Constraint

In this section we will remove the gradient constraint from the variational solutions constructed in Proposition 5.1. This will be achieved by showing that the gradient is bounded in terms of a constant depending only on the data. Throughout this section we assume all hypothesis of Theorem 1.3 to be in force.

### Properties of the Integrand Function *f*

The following proposition contains some useful properties of *f* and its polar function (convex conjugate) $f^*:\mathbb {R}^n\rightarrow [-\infty ,\infty ]$ defined by

$$\begin{aligned} f^*(\eta ):=\sup _{\xi \in \mathbb {R}^n}\big \{ \eta \cdot \xi -f(\xi )\big \}. \end{aligned}$$

#### Proposition 6.1

Suppose that $f:\mathbb {R}^n\rightarrow \mathbb {R}$ satisfies the set of assumptions (A2). Then, we have

1. there exists $c=c(\varepsilon )\in \mathbb {R}$ such that
  $$\begin{aligned} f(\xi )\ge \tfrac{1}{4} \varepsilon |\xi |^2 - c \quad \text{ for } \text{ any } \xi \in \mathbb {R}^n; \end{aligned}$$
2. $f^*$ is superlinear and at most of quadratic qrowth, i.e. such that
  $$\begin{aligned} f^*(\eta )\le \tfrac{2}{\varepsilon }|\eta |^2 + c \quad \text{ for } \text{ any } \eta \in \mathbb {R}^n; \end{aligned}$$
3. there exists $r>0$ such that $f^*\in C^{1,1}(\mathbb {R}^n\setminus B_r(0))$ and
  $$ |D^2 f^*(\eta )|\le \tfrac{1}{\varepsilon } \quad \text{ for } \text{ a.e. } \eta \in \mathbb {R}^n\setminus B_r(0); $$
4. the restriction of $\nabla f$ to $\mathbb {R}^n\setminus B_1$ is an invertible vector-field with
  $$\begin{aligned} (\nabla f)^{-1}=\nabla f^*. \end{aligned}$$

#### Proof

Property (1) follows for large values of $|\xi |$ by Taylor’s expansion and the uniform convexity of *f* from (1.7). Property (2) is a straightforward consequence of the definition of $f^*$ taking into account that *f* is super-linear (cf. [20, Lemma 3.1]) and the assertion from (1). The proof of property (3) can be found in [19] for a special class of functions. The general case can be retrieved from [20, Lemma 3.3]. Property (4) is a consequence of [18, Chapter 1, Corollary 5.2] and the fact that *f* is a $C^2$-function outside the unit ball by assumption. $\square$

### Construction of Barriers

Let $\alpha \in \mathbb {R}\setminus \{0\}$. For $y:[0,T]\rightarrow \mathbb {R}^n$ and $c:[0,T]\rightarrow \mathbb {R}$ we consider the following function:

6.1 $$\begin{aligned} v(x,t) = \frac{n}{\alpha }f^*\Big (\frac{\alpha }{n}\big (x-y(t)\big )\Big )-c(t). \end{aligned}$$

In view of Proposition 6.1 (3), (4) it is easy to check that

6.2 $$\begin{aligned} {{\,\textrm{div}\,}}_x \nabla _\xi f\big (\nabla v(x,t)\big )=\alpha , \end{aligned}$$

provided that

$$\begin{aligned} \Big |\frac{\alpha }{n}\big (x-y(t)\big )\Big | \ge r \quad \text{ and }\quad \Big |\nabla f^*\Big (\frac{\alpha }{n}\big (x-y(t)\big )\Big )\Big | \ge 1. \end{aligned}$$

We recall that the class of functions in (6.1) was introduced by Cellina in [14] for the stationary case. In the same paper it was also shown that this class of functions has a property corresponding to (6.2) at the level of the associated variational integral, i.e. they minimize the variational integral that formally has (6.2) as Euler-Lagrange equation. For this, it was only necessary to assume that *f* is convex. Our goal is to show that for suitable choices of $\alpha$, *y*(*t*) and *c*(*t*) the function *v* can be used in the time dependent setting as a barrier function. We start with an auxiliary lemma.

#### Lemma 6.2

Let $\alpha \in \mathbb {R}\setminus \{0\}$ and $M,Q_1>0$. For $b\in \mathbb {R}$ and $w\in \mathbb {R}^n$ we define

$$ \widetilde{\Omega }_{b,w} := \left\{ x\in \mathbb {R}^n: \frac{n}{\alpha }f^*\left( \frac{\alpha }{n}\,x \right) -w\cdot x-b\le 0\right\} . $$

If

$$ b > \min _{x\in \mathbb {R}^n} \Big [ \frac{n}{\alpha }f^*\left( \frac{\alpha }{n}\,x \right) -w\cdot x\Big ], $$

then $\widetilde{\Omega }_{b,w}$ is non-empty, bounded and convex. Furthermore, if

6.3 $$\begin{aligned} b \ge \Gamma := \max _{x\in \overline{B_M(0)},\,w\in \overline{B_{Q_1}(0)} }\Big [ \frac{n}{\alpha }f^*\left( \frac{\alpha }{n}\,x \right) -w\cdot x\Big ], \end{aligned}$$

then, for any $w\in \overline{B_{Q_1}(0)}$ the ball $\overline{B_M(0)}$ is contained in $\widetilde{\Omega }_{b,w}$. $\square$

#### Proof

The proof is straightforward. For the sake of completeness, however, we will elaborate on it. Since $f^*$ has superlinear growth and $\mathbb {R}^n\ni x\mapsto w\cdot x$ is linear, we have

$$ k(x):=\frac{n}{\alpha }f^*\left( \frac{\alpha }{n}\,x \right) -w\cdot x\rightarrow \infty \quad \text{ as } |x|\rightarrow \infty . $$

This implies that *k* attains its minimum on $\mathbb {R}^n$. If *b* is larger than the minimal value of *k*, we obtain that $\widetilde{\Omega }_{b,w}$ is non-empty and bounded. In addition, $\widetilde{\Omega }_{b,w}$ is convex because it is constructed as a sublevel set of a convex function. This proves the first assertion of the lemma.

The second assertion, i.e. that (6.3) implies $\overline{B_M(0)}\subset \widetilde{\Omega }_{b,w}$, follows similarly. Fix $w\in \overline{B_{Q_1}(0)}$. Then *k* is bounded on $\overline{B_M(0)}$. If *b* is greater than the maximum value of *k* on $\overline{B_M(0)}$, then $\overline{B_M(0)}$ is a subset of $\widetilde{\Omega }_{b,w}$, proving the second claim. $\square$

#### Proposition 6.3

Let the assumptions of Theorem 1.3 be in force. Then, there exists $\alpha _o>0$ depending only on $n,\varepsilon , {{\,\textrm{diam}\,}}(\Omega ), \Vert D^2f\Vert _{L^\infty (\mathbb {R}^n\setminus B_1)}, \Vert \partial _t g\Vert _\infty , \mathsf Q$ such that for any $\alpha \in \mathbb {R}$ with $|\alpha |\ge \alpha _o$ and any $x_o\in \partial \Omega$ the functions *y*(*t*) and *c*(*t*) can be chosen in such a way that the function *v* defined in (6.1) satisfies the following properties:

1. $$v(x_o,t)=g(x_o,t)$$ for any $$t\in [0,T)$$;
2. $v(x,t)\le g(x,t)$ for any $x\in \Omega$ and $t\in [0,T)$ if $\alpha >0$; if $\alpha <0$ the converse inequality holds true;
3. if $\alpha >0$ is large enough, then *v* is a sub-solution to the parabolic equation (1.3); similarly, if $\alpha <0$ is chosen small enough, then *v* is a super-solution.
4. $v\in \textrm{Lip}^x(\Omega _T)$ with Lipschitz constant *L* depending only on the data *n*, $\varepsilon$, *R*, ${{\,\textrm{diam}\,}}(\Omega )$, *f*, $\nabla f$, *Q*, $\Vert \nabla g\Vert _{L^\infty (\Omega _T,\mathbb {R}^n)}$, and $\alpha$.

Note that for the purposes of what follows, it would be sufficient for condition (2) to hold on the parabolic boundary $\partial _{\mathcal {P}}\Omega _T$. However, the proof establishes the stronger result that condition (2) is satisfied throughout the entire cylinder $\Omega _T$.

#### Proof

We only prove the assertions for positive $\alpha$, since the proof for the other case is analogous. We may assume $\alpha \ge \alpha _o\ge n$ by choosing $\alpha _o\ge n$.

*Step 1: An auxiliary set.* Let *R* be the radius from the *R*-uniformly convexity assumption (A1) on $\Omega$ and $\nu _{x_o}$ the outward pointing unit vector associated with $x_o\in \partial \Omega$; see Definition 2.1. From Remark 2.2 we conclude that $\Omega \subset B_R(x_o-R\nu _{x_o})$.

Next, we let $w_{x_o}^-:[0,T]\rightarrow \mathbb {R}^n$ be the function from the $t-\textrm{BSC}_Q$ condition in (A3); see Definition 2.3. By $\widetilde{w}_{x_o}^- :[0,T]\rightarrow \mathbb {R}^n$ we denote the modified function constructed in Lemma 2.5 satisfying $\Vert \widetilde{w}_{x_o}^-\Vert _{L^\infty ([0,T],\mathbb {R}^n)}\le Q_1:=Q+ \Vert \nabla g\Vert _{L^\infty (\Omega _T,\mathbb {R}^n)}$ and $\Vert (\widetilde{w}_{x_o}^-)'\Vert _{L^\infty ([0,T],\mathbb {R}^n)} = \Vert (w_{x_o}^-)'\Vert _{L^\infty ([0,T],\mathbb {R}^n)}\le \mathsf Q$ and such that

6.4 $$\begin{aligned} \widetilde{g}(x,t) := g(x_o,t)+\widetilde{w}_{x_o}^{-}(t)\cdot (x-x_o) \le g(x,t) \end{aligned}$$

for any $(x,t)\in \overline{\Omega }\times [0,T)$. For any $t\in [0,T)$ we define the set

$$\begin{aligned} \widetilde{\Omega }_t := \bigg \{x\in \mathbb {R}^n: \underbrace{\frac{n}{\alpha }f^*\Big (\frac{\alpha }{n}\big (x-y(t)\big )\Big )-c(t)}_{=\, v(x,t)}\le \widetilde{g}(x,t)\bigg \}. \end{aligned}$$

*Step 2: Proof of claim (1).* Here we choose *y*(*t*) and *c*(*t*) in such a way that

6.5 $$\begin{aligned} x_o\in \partial \widetilde{\Omega }_t \quad \text{ for } \text{ any } t\in [0,T). \end{aligned}$$

In view of the definition of $\widetilde{\Omega }_t$ this implies claim (1).

By $\varepsilon$ and *r* we denote the corresponding parameters from (1.7) and Proposition 6.1 (3). Due to the super-linearity of $f^*$ there exists $M\ge r+{{\,\textrm{diam}\,}}(\Omega )$, depending only on $\varepsilon , R, {{\,\textrm{diam}\,}}(\Omega ), \nabla f, Q_1, \alpha$, such that

6.6 $$\begin{aligned} |\nabla f^*(\eta )|>\frac{\alpha R}{\varepsilon n}+Q_1\qquad \text{ for } \text{ any } \eta \in \mathbb {R}^n\setminus B_{M}(0). \end{aligned}$$

We define $\Gamma$ according to (6.3) from Lemma 6.2 for this particular choice of *M*. Note that $\Gamma$ depends on $n, \varepsilon , R, {{\,\textrm{diam}\,}}(\Omega ), \nabla f, Q_1, \alpha$. Again, due to the super-linearity of $f^*$ and the upper bound $|\widetilde{w}_{x_o}^-(t)|\le Q_1$ for any $t\in [0,T)$ there exists $\varrho _o\ge M$ such that

6.7 $$\begin{aligned} B(t,\eta ) := \frac{n}{\alpha }f^*\left( \frac{\alpha }{n}\,\eta \right) - \widetilde{w}_{x_o}^-(t)\cdot \eta \ge \Gamma \quad \text{ for } \text{ any } \eta \in \mathbb {R}^n\setminus B_{\varrho _o} \text{ and } t\in [0,T). \end{aligned}$$

Note that $\varrho _o$ can be chosen in dependence on $n, \varepsilon , R, {{\,\textrm{diam}\,}}(\Omega ), f, \nabla f, Q_1, \alpha$. Next we fix $\lambda >Q_1+\max \{1, r\}$ such that

6.8 $$\begin{aligned} z_\lambda (t):=\frac{n}{\alpha }\nabla f\big (\widetilde{w}_{x_o}^-(t)+\lambda \nu _{x_o}\big ) \in \mathbb {R}^n\setminus B_{\varrho _o} \quad \text{ for } \text{ any } t\in [0,T). \end{aligned}$$

This can be achieved, because $|\widetilde{w}_{x_o}^-(t)|\le Q_1$ and since *f* has super-linear growth. Note that $\lambda$ can be chosen in dependence on $n, \varepsilon , R, {{\,\textrm{diam}\,}}(\Omega ), f, \nabla f, Q_1, \alpha$. Moreover, $|\widetilde{w}_{x_o}^-(t)+\lambda \nu _{x_o}|\ge \lambda -Q_1>1$, which ensures that the gradient in (6.8) is well defined. Using Lemma 6.1 (4), i.e. the fact that $(\nabla f)^{-1}=\nabla f^*$ on $\mathbb {R}^n\setminus B_1$, (6.8) can be re-written in the form

6.9 $$\begin{aligned} \nabla f^*\left( \frac{\alpha }{n}\,z_\lambda (t)\right) -\widetilde{w}_{x_o}^-(t)=\lambda \nu _{x_o}. \end{aligned}$$

Note that $|\frac{\alpha }{n}z_\lambda (t)|\ge |z_\lambda (t)|\ge \varrho _o\ge M>r$, so that by Lemma 6.1 (3), $\nabla f^*\left( \frac{\alpha }{n}\,z_\lambda (t)\right)$ is well defined. Next, we let

$$\begin{aligned} b(t) := B(t,z_\lambda (t)) = \frac{n}{\alpha }f^*\left( \frac{\alpha }{n}\,z_\lambda (t) \right) - \widetilde{w}_{x_o}^-(t)\cdot z_\lambda (t) \quad \text{ for } t\in [0,T), \end{aligned}$$

and observe that (6.8) and (6.7) imply

6.10 $$\begin{aligned} b(t) \ge \Gamma \quad \text{ for } \text{ any } t\in [0,T). \end{aligned}$$

For any $t\in [0,T)$ we now define the set

6.11 $$\begin{aligned} \widetilde{\Omega }_{b(t),\widetilde{w}_{x_o}^-(t)}=\Big \{ x\in \mathbb {R}^n:\frac{n}{\alpha }f^*\left( \frac{\alpha }{n}\,x \right) -\widetilde{w}_{x_o}^-(t)\cdot x-b(t)\le 0 \Big \}. \end{aligned}$$

By definition we have $z_\lambda (t)\in \partial \widetilde{\Omega }_{b(t),\widetilde{w}_{x_o}^-(t)}$ for any $t\in [0,T)$. Moreover, (6.9) implies that the sets $\widetilde{\Omega }_{b(t),\widetilde{w}_{x_o}^-(t)}$ have outward normal $\nu _{x_o}$ at $z_\lambda (t)\in \partial \widetilde{\Omega }_{b(t),\widetilde{w}_{x_o}^-(t)}$ for any $t\in [0,T)$. Finally, we define

6.12 $$\begin{aligned} y(t):=x_o-z_\lambda (t), \end{aligned}$$

and

6.13 $$\begin{aligned} c(t)&:= b(t) + \widetilde{w}_{x_o}^-(t)\cdot z_\lambda (t) -g(x_o,t) \nonumber \\&\ = \frac{n}{\alpha }f^*\left( \frac{\alpha }{n}\big (x_o-y(t)\big )\right) -g(x_o,t) \end{aligned}$$

and observe that

$$ \widetilde{\Omega }_t = y(t)+\widetilde{\Omega }_{b(t),\widetilde{w}_{x_o}^-(t)}. $$

Since $z_\lambda (t)\in \partial \widetilde{\Omega }_{b(t),\widetilde{w}_{x_o}^-(t)}$, we have $x_o\in \partial \widetilde{\Omega }_t$ for any $t\in [0,T)$, which shows (6.5). Moreover, $\widetilde{\Omega }_t$ has outward normal $\nu _{x_o}$ at $x_o\in \partial \widetilde{\Omega }_t$ for any $t\in [0,T)$.

*Step 3: Proof of claim (2).* Here we prove that

6.14 $$\begin{aligned} \Omega \subset \widetilde{\Omega }_t \quad \text{ for } \text{ any } t\in [0,T). \end{aligned}$$

The definition of $\widetilde{\Omega }_t$ and (6.4) imply claim (2).

We fix a time $t\in [0,T)$ and abbreviate $b=b(t)$ and $w=\widetilde{w}_{x_o}^-(t)$. With these abbreviations the set in (6.11) can be re-written as

$$\begin{aligned} \widetilde{\Omega }_{b,w}=\Big \{ x\in \mathbb {R}^n:\frac{n}{\alpha }f^*\left( \frac{\alpha }{n}\,x \right) -w\cdot x-b\le 0 \Big \}. \end{aligned}$$

Since $|w|\le Q_1$ and $b\ge \Gamma$ by (6.10), Lemma 6.2 ensures that $\overline{B_{M}(0)}\subset \widetilde{\Omega }_{b,w}$, so that $|x|\ge M\ge r+{{\,\textrm{diam}\,}}(\Omega )$ for any $x\in \partial \widetilde{\Omega }_{b,w}$. Noting that $\alpha \ge \alpha _o\ge n$ we conclude

6.15 $$\begin{aligned} \big |\tfrac{\alpha }{n}x\big |\ge M\ge r+{{\,\textrm{diam}\,}}(\Omega ), \qquad \text{ for } \text{ any } x\in \partial \widetilde{\Omega }_{b,w}. \end{aligned}$$

Hence, Proposition 6.1 (3) ensures that $f^*$ is of class $C^{1,1}$ in a neighborhood of $\frac{\alpha }{n}x$ whenever $x\in \partial \widetilde{\Omega }_{b,w}$. Next, we compute the principal curvatures of $\partial \widetilde{\Omega }_{b,w}$. To this aim we need to know that for any $x\in \partial \widetilde{\Omega }_{b,w}$ there holds

$$ \nabla f^*\Big (\frac{\alpha }{n}\,x\Big ) - w \not = 0. $$

Indeed, due to (6.15) and (6.6), we have

6.16 $$\begin{aligned} \min _{x\in \partial \widetilde{\Omega }_{b,w}} \Big |\nabla f^*\Big (\frac{\alpha }{n}\,x\Big ) - w\Big |>\frac{\alpha R}{\varepsilon n}. \end{aligned}$$

Consequently, for $x\in \partial \widetilde{\Omega }_{b,w}$ the outward pointing normal $\widetilde{\nu }_x$ to $\partial \widetilde{\Omega }_{b,w}$ in *x* is given by

$$ \widetilde{\nu }_x =\frac{\nabla f^*\big (\frac{\alpha }{n}x\big ) - w}{\big |\nabla f^*\big (\frac{\alpha }{n}x\big ) - w\big |}, $$

and the tangent space of $\partial \widetilde{\Omega }_{b,w}$ at *x* is given by $T_x\partial \widetilde{\Omega }_{b,w}= (\widetilde{\nu }_x\mathbb {R})^\perp$. The second fundamental form $\widetilde{A}_x:T_x\partial \widetilde{\Omega }_{b,w}\times T_x\partial \widetilde{\Omega }_{b,w}\rightarrow \mathbb {R}$ of $\partial \widetilde{\Omega }_{b,w}$ at *x* is given by

6.17 $$\begin{aligned} \widetilde{A}_x(\xi ,\zeta ) =-\frac{\alpha }{n} \frac{D^2f^*\big (\frac{\alpha }{n}x\big )(\xi ,\zeta )}{\big |\nabla f^*\big (\frac{\alpha }{n}x\big ) - w\big |}, \qquad \forall \,\xi ,\eta \in T_x\partial \widetilde{\Omega }_{b,w}, \end{aligned}$$

provided $D^2f^*\big (\frac{\alpha }{n} x\big )$ exists. This, however, cannot be guaranteed in general. Therefore, we consider $\widetilde{\Omega }_{\tau ,w}$ with $\tau \in [b,b+\delta )$ instead of $\widetilde{\Omega }_{b,w}$ and show by a co-area formula type argument the existence of second derivatives $\mathcal {H}^{n-1}$-a.e. on $\partial \widetilde{\Omega }_{\tau ,w}$ for a.e. $\tau \in [b,b+\delta )$. Indeed, using the fact that $\partial \widetilde{\Omega }_{\tau ,w}$ is the level set $h_{w}^{-1} \{ \tau \}$ of the function $h_{w}(x):= \frac{n}{\alpha } f^*\big (\frac{\alpha }{n}x\big ) -w\cdot x$, the co-area formula implies for any $\delta >0$ that

$$\begin{aligned} \int _{b}^{b+\delta } \mathcal {H}^{n-1}\big ( h_{w}^{-1} \{ \tau \}\cap \Sigma \big )\, \textrm{d}\tau&= \int _{ (\widetilde{\Omega }_{w,b+\delta }\setminus \widetilde{\Omega }_{b,w})\cap \Sigma }|\nabla h_{w}(x)|\, \textrm{d}x\\&= \int _{ (\widetilde{\Omega }_{w,b+\delta }\setminus \widetilde{\Omega }_{b,w})\cap \Sigma }\big |\nabla f^*\big (\tfrac{\alpha }{n}x\big )-w\big |\, \textrm{d}x, \end{aligned}$$

where $\Sigma$ denotes the set of points in which $D^2f^*(\frac{\alpha }{n}x)$ does not exist. This, however, is a set of $\mathcal {L}^n$-measure zero by Rademacher’s theorem, and therefore the right-hand side above is equal to zero. This proves that $\mathcal {H}^{n-1}( h_{w}^{-1} \{ \tau \}\cap \Sigma )=0$ for a.e. $\tau \in [b,b+\delta )$. Therefore we can choose a sequence $(\tau _i)_{i\in \mathbb {N}}\subset [b,b+\delta )$ such that $\tau _i\downarrow b$ and $D^2f^*(\frac{\alpha }{n}x)$ exists for $\mathcal {H}^{n-1}$-almost every $x\in \partial \widetilde{\Omega }_{\tau _i, w}$. Due to the continuity of $f^*$ and $\nabla f^*$ in a neighborhood of $\frac{\alpha }{n}(x_o-y(t))$ there exist $x_i\in \mathbb {R}^n$ and $\nu _i\in \mathbb {R}^n$ such that $x_i\in y(t)+\partial \widetilde{\Omega }_{\tau _i, w}$, $\nu _i$ is the outward pointing normal of $y(t)+\partial \widetilde{\Omega }_{\tau _i, w}$ at $x_i$, $x_i\rightarrow x_o$ and $\nu _i\rightarrow \nu _{x_o}$ as $i\rightarrow \infty$. Using Proposition 6.1 (3) and (6.16) to estimate the right-hand side in (6.17), we obtain for $\mathcal {H}^{n-1}$-a.e. $x\in \partial \widetilde{\Omega }_{\tau _i,w}$ that

6.18 $$\begin{aligned} \widetilde{A}_x(\xi ,\xi )&=- \frac{\alpha }{n} \frac{D^2f^*\big (\frac{\alpha }{n}x\big )(\xi ,\xi )}{\big |\nabla f^*\big (\frac{\alpha }{n}x\big )- w\big |} \le \frac{\alpha }{n} \frac{\frac{1}{\varepsilon }|\xi |^2}{\frac{\alpha R}{\varepsilon n}} = \tfrac{1}{R} |\xi |^2 \quad \forall \, \xi \in \mathbb {R}^n. \end{aligned}$$

This ensures that for $\mathcal {H}^{n-1}$-a.e. $x\in \partial \widetilde{\Omega }_{\tau _i,w}$ the principal curvatures of $\partial \widetilde{\Omega }_{\tau _i,w}$ are smaller than $\frac{1}{R}$, which is the principal curvature of $B_R(x_i-R\nu _{i})$. By the convexity of $\widetilde{\Omega }_{\tau _i,w}$ and the fact that $f^*$ is of class $C^{1,1}$ this implies $B_R(x_i-R\nu _{i})\subset y(t)+\widetilde{\Omega }_{\tau _i,w}$ for any $i\in \mathbb {N}$. Passing to the limit $i\rightarrow \infty$, we conclude $B_R(x_o-R\nu _{x_o})\subset y(t)+\widetilde{\Omega }_{b,w}=\widetilde{\Omega }_t$, which in view of Remark 2.2 implies the set inclusion (6.14).

*Step 4: Proof of claim (3).* In this step we will choose $\alpha _o$ in such a way that *v* is a sub-solution to (1.3) whenever $\alpha \ge \alpha _0$. We compute

$$\begin{aligned} \partial _t v(x,t)&= \partial _t \Big [\frac{n}{\alpha }f^*\left( \frac{\alpha }{n}\big (x-y(t)\big )\right) -c(t)\Big ] \\&= -\Big [\nabla f^*\left( \frac{\alpha }{n}\big (x-y(t)\big )\right) -\nabla f^*\left( \frac{\alpha }{n}\big (x_o-y(t)\big )\right) \Big ]\cdot y'(t) + \partial _t g(x_o,t). \end{aligned}$$

From (6.8) and the choices of $\rho _o$ and *M* we know that

$$\begin{aligned} |x_o-y(t)| = |z_\lambda (t)| \ge \rho _o \ge M \ge r+{{\,\textrm{diam}\,}}(\Omega ). \end{aligned}$$

Since $\alpha \ge \alpha _o\ge n$ this ensures $\frac{\alpha }{n}(x-y(t))\in \mathbb {R}^n\setminus B_r$ for any $x\in \Omega$. Since $\Omega$ is convex, we thus have that $\frac{\alpha }{n}(\xi -y(t))\in \mathbb {R}^n\setminus B_r$ for any $\xi \in [x,x_o]$. Therefore, we may use Proposition 6.1 (3) to conclude that

$$\begin{aligned} |\partial _t v(x,t)|&\le \frac{1}{\varepsilon } \frac{\alpha }{n}|x-x_o| |y'(t)| + \Vert \partial _t g\Vert _{L^\infty (\Omega _T)} \\&= \frac{1}{\varepsilon } \frac{\alpha }{n}|x-x_o| |z_\lambda '(t)| + \Vert \partial _t g\Vert _{L^\infty (\Omega _T)} \\&\le \frac{1}{\varepsilon } {{\,\textrm{diam}\,}}(\Omega ) \big |D^2f\big (\widetilde{w}_{x_o}^-(t)+\lambda \nu _{x_o}\big )\big | |(\widetilde{w}_{x_o}^-)'(t)| + \Vert \partial _t g\Vert _{L^\infty (\Omega _T)} . \end{aligned}$$

Note that the choice of $\lambda$ ensures that $|\widetilde{w}_{x_o}^-(t)+\lambda \nu _{x_o}|\ge \lambda - Q_1\ge 1$ and hence $\widetilde{w}_{x_o}^-(t)+\lambda \nu _{x_o}\in \mathbb {R}^n\setminus B_1$. Since $D^2f$ is bounded outside the unit ball by assumption (A2) and $|(\widetilde{w}_{x_o}^-)'(t)|\le \mathsf Q$ by (A3) we finally conclude that

$$\begin{aligned} |\partial _t v(x,t)|&\le \frac{1}{\varepsilon }{{\,\textrm{diam}\,}}(\Omega ) \Vert D^2f\Vert _{L^\infty (\mathbb {R}^n\setminus B_1)}\mathsf Q + \Vert \partial _t g\Vert _{L^\infty (\Omega _T)}. \end{aligned}$$

We choose that

6.19 $$\begin{aligned} \alpha _o := \max \bigg \{n \,, \, \frac{1}{\varepsilon } {{\,\textrm{diam}\,}}(\Omega ) \Vert D^2f\Vert _{L^\infty (\mathbb {R}^n\setminus B_1)} \mathsf Q+ \Vert \partial _t g\Vert _{L^\infty (\Omega _T)} \bigg \}. \end{aligned}$$

Note that $\alpha _o$ depends on $n,\varepsilon , {{\,\textrm{diam}\,}}(\Omega ), \Vert D^2f\Vert _{L^\infty (\mathbb {R}^n\setminus B_1)}, \Vert \partial _t g\Vert _{L^\infty (\Omega _T)}$ and $\mathsf Q$. In view of the preceding computation and (6.2) we have for any $\alpha \ge \alpha _o$ that

6.20 $$\begin{aligned} \partial _t v(x,t)-{{\,\textrm{div}\,}}\nabla f\big (\nabla v(x,t)\big )\le |\partial _t v(x,t)|-\alpha \le 0 , \end{aligned}$$

i.e. *v* is sub-solution of the parabolic equation (1.3) and hence claim (3) is proved.

*Step 5: Proof of claim (4).* With (6.12) and (6.9) we compute that

$$\begin{aligned} \nabla v(x,t)&= \nabla f^*\left( \frac{\alpha }{n}\big (x-y(t)\big )\right) \\&= \nabla f^*\left( \frac{\alpha }{n}\big (x-y(t)\big )\right) - \nabla f^*\left( \frac{\alpha }{n}\big (x_o-y(t)\big )\right) + \nabla f^*\Big (\frac{\alpha }{n}z_\lambda (t)\Big ) \\&= \nabla f^*\left( \frac{\alpha }{n}\big (x-y(t)\big )\right) - \nabla f^*\left( \frac{\alpha }{n}\big (x_o-y(t)\big )\right) + \lambda \nu _{x_o} + \widetilde{w}_{x_o}^-(t). \end{aligned}$$

The difference of the first two terms is bounded exactly as in Step 4, so that

$$\begin{aligned} |\nabla v(x,t)|&\le \frac{\alpha }{\varepsilon n}{{\,\textrm{diam}\,}}(\Omega ) + \lambda +\Vert \widetilde{w}_{x_o}^{-}\Vert _{L^\infty ([0,T],\mathbb {R}^n)} \\&\le \frac{\alpha }{\varepsilon n}{{\,\textrm{diam}\,}}(\Omega ) + \lambda + Q_1. \end{aligned}$$

This implies

$$\begin{aligned} |\nabla v(x,t)|&\le c\big (n, \varepsilon , R, {{\,\textrm{diam}\,}}(\Omega ), f, \nabla f, Q_1, \alpha \big ). \end{aligned}$$

This proves claim (4) and finishes the proof of Proposition 6.3. $\square$

### Parabolic Sub- Super-Minimizers and the Comparison Principle

We recall that the variational solution constructed in Proposition 4.1 admits a time derivative $\partial _tu\in L^2(\Omega _T)$. Therefore, we may perform an integration by parts in the first term on the right-hand side of the variational inequality (1.6). In this way, the variational inequality can be re-written as

$$\begin{aligned} \iint _{\Omega _T}f(\nabla u)\,\textrm{d}x\textrm{d}t&\le \iint _{\Omega _T}\big [\partial _{t}u(v-u) + f(\nabla v)\big ]\,\textrm{d}x\textrm{d}t\end{aligned}$$

for any $v\in \textrm{Lip}^x_g(\Omega _T, L)$ with $\partial _{t}v\in L^{2}(\Omega _{T})$. Note that the assumption $\partial _{t}v\in L^{2}(\Omega _{T})$ can be eliminated by an approximation argument. This motivates the following definition:

#### Definition 6.4

*(Parabolic sub-/super-minimizer)* Let $L\in (0,\infty ]$. A map $u\in \textrm{Lip}^x(\Omega _T, L)$ with $\partial _t u\in L^2(\Omega _T)$ is called *parabolic sub-minimizer (of the gradient constrained obstacle problem in the case* $L<\infty$*)* in $\textrm{Lip}^x(\Omega _T, L)$ if and only if

6.21 $$\begin{aligned} \iint _{\Omega _T}f(\nabla u)\,\textrm{d}x\textrm{d}t&\le \iint _{\Omega _T}\big [\partial _{t}u(v-u) + f(\nabla v)\big ]\,\textrm{d}x\textrm{d}t\end{aligned}$$

holds true for any $v\in \textrm{Lip}^x_u(\Omega _T, L)$ with $v\le u$ in $\Omega _T$. Moreover, a map $u\in \textrm{Lip}^x(\Omega _T, L)$ is called *parabolic super-minimizer* in $\textrm{Lip}^x(\Omega _T, L)$ if and only if (6.21) holds true for any $v\in \textrm{Lip}^x_u(\Omega _T, L)$ with $v\ge u$ in $\Omega _T$. Finally, $u\in \textrm{Lip}^x(\Omega _T, L)$ is called *parabolic minimizer* if and only if (6.21) holds true for any $v\in \textrm{Lip}^x_u(\Omega _T, L)$.

The concept of parabolic minimizers for vector-valued integrands with quadratic growth originates from the work of Wieser [43]. Subsequently, it will be essential to establish that a localization principle with respect to the spatial variables holds for parabolic minimizers

#### Remark 6.5

*(Localization in space)* Let $L\in (0,\infty )$ and suppose that $\Omega '\subset \Omega$ is an open, convex set, $f:\mathbb {R}^n\rightarrow \mathbb {R}$ a convex integrand and $u\in \textrm{Lip}^x(\Omega _T, L)$ with $\partial _t u\in L^2(\Omega _T)$ a parabolic minimizer in the sense of Definition 6.4 in $\Omega _T$. Then, *u* is also a parabolic minimizer in the subcylinder $\Omega '_T:=\Omega '\times [0,T)$. The proof of this elementary fact is analogous to the setting of time independent boundary data and can be found in [6, Remark 4.2].

In the next lemma, we establish the comparison principle for parabolic sub- and super-minimizers.

#### Lemma 6.6

(Comparison principle) Let $L\in (0,\infty ]$ and suppose that $\Omega \subset \mathbb {R}^n$ is a bounded open set, $f:\mathbb {R}^n\rightarrow \mathbb {R}$ is a convex integrand and $u, \tilde{u}\in \textrm{Lip}^x(\Omega _T, L)$ with $\partial _t u,\partial _t\tilde{u}\in L^2(\Omega _T)$. Suppose that *u* is a sub-minimizer and $\tilde{u}$ is a super-minimizer in $\Omega _T$ in the sense of Definition 6.4 and that $u\le \tilde{u}$ on $\partial _{\mathcal {P}}\Omega _T$. Then, we have

$$ u\le \tilde{u}\quad \text{ a.e. } \text{ in } \Omega _T. $$

#### Proof

Let $\tau \in (0,T]$. We define that

$$\begin{aligned} v:= \left\{ \begin{array}{cl} \min \{u, \tilde{u}\}, &  \text{ in } \Omega _\tau , \\ u, &  \text{ in } \Omega \times [\tau ,T), \end{array}\right. \end{aligned}$$

and

$$\begin{aligned} w:= \left\{ \begin{array}{cl} \max \{u, \tilde{u}\}, &  \text{ in } \Omega _\tau , \\ \tilde{u}, &  \text{ in } \Omega \times [\tau ,T). \end{array}\right. \end{aligned}$$

We note that $v\in \textrm{Lip}^x_u(\Omega _T, L)$ with $v\le u$ and $v(0)=u_o$ and $w\in \textrm{Lip}^x_{\tilde{u}}(\Omega _T, L)$ with $w\ge \tilde{u}$ and $w(0)=\tilde{u}_o$. This ensures that *v* is an admissible comparison function in the variational inequality (6.21) for *u* and *w* for of $\tilde{u}$. Adding the two resulting inequalities and taking into account that the parts of the integrals on $\Omega \times (\tau ,T)$ cancel themselves out, we obtain

6.22 $$\begin{aligned}&\iint _{\Omega _\tau }\big [f(\nabla u) + f(\nabla \tilde{u})\big ] \,\textrm{d}x\textrm{d}t\nonumber \\&\qquad \le \iint _{\Omega _\tau }\big [f(\nabla v) + f(\nabla w) + \partial _t u (v-u) + \partial _t \tilde{u}(w-\tilde{u})\big ]\,\textrm{d}x\textrm{d}t. \end{aligned}$$

We now consider the terms on the right-hand side of (6.22). From the definition of *v* and *w* we infer

$$\begin{aligned} \iint _{\Omega _\tau }\big [f(\nabla v) + f(\nabla w)\big ] \,\textrm{d}x\textrm{d}t= \iint _{\Omega _\tau }\big [f(\nabla u) + f(\nabla \tilde{u})\big ] \,\textrm{d}x\textrm{d}t. \end{aligned}$$

Moreover, we observe that $v-u=-(u-\tilde{u})_+$ and $w-\tilde{u}=(u-\tilde{u})_+$ in $\Omega _\tau$, so that

$$\begin{aligned} \partial _t u(v-u) + \partial _t \tilde{u}(w-\tilde{u})&= -\partial _t (u-\tilde{u})(u-\tilde{u})_+ = -\tfrac{1}{2}\partial _t (u-\tilde{u})_+^2 . \end{aligned}$$

This implies

$$\begin{aligned} \iint _{\Omega _\tau } \big [\partial _t u(v-u) + \partial _t \tilde{u}(w-\tilde{u})\big ] \textrm{d}x\textrm{d}t&= -\tfrac{1}{2}\iint _{\Omega _\tau } \partial _t (u-\tilde{u})_+^2 \textrm{d}x\textrm{d}t\\&= -\tfrac{1}{2} \int _{\Omega \times \{\tau \}} (u-\tilde{u})_+^2 \textrm{d}x. \end{aligned}$$

Here we used the assumption that $(u-\tilde{u})_+(0)=0$. Joining the preceding identities with (6.22), we conclude

$$\begin{aligned} \int _{\Omega \times \{\tau \}}(u - \tilde{u})_+^2\textrm{d}x\le 0 . \end{aligned}$$

Since $\tau \in (0,T]$ was arbitrary, this proves the claim $u\le \tilde{u}$ a.e. in $\Omega _T$. $\square$

As a consequence of the preceding comparison principle, we obtain the following result:

#### Lemma 6.7

(Maximum principle) Let $L\in (0,\infty ]$ and suppose that $\Omega \subset \mathbb {R}^n$ is open and bounded, $f:\mathbb {R}^n\rightarrow \mathbb {R}$ convex and let $u, \tilde{u}\in \textrm{Lip}^x(\Omega _T, L)$ with $\partial _t u,\partial _t\tilde{u}\in L^2(\Omega _T)$. Suppose that *u* is a sub-minimizer and $\tilde{u}$ is a super-minimizer in the sense of Definition 6.4 in $\Omega _T$. Then, we have

$$ \sup _{\Omega _T}(u-\tilde{u})= \sup _{\partial _{\mathcal {P}}\Omega _{T}}(u-\tilde{u}). $$

#### Proof

For $(x,t)\in \partial _{\mathcal {P}}\Omega _T$ it holds that

$$ u(x,t) = \tilde{u}(x,t) + u(x,t) - \tilde{u}(x,t) \le \tilde{u}(x,t) + \sup _{\partial _{\mathcal {P}}\Omega _T}(u - \tilde{u}). $$

Since $\tilde{u}$ is a parabolic super-minimizer in $\Omega _T$ it immediately follows that also $\tilde{u}+\sup _{\partial _{\mathcal {P}}\Omega _T}(u - \tilde{u})$ is parabolic super-minimizer in $\Omega _T$. In view of Lemma 6.6 we therefore have

$$ u \le \tilde{u}+\sup _{\partial _{\mathcal {P}}\Omega _T}(u - \tilde{u}) \quad \text{ in } \Omega _T. $$

This inequality can be re-written in the form

$$ \sup _{\Omega _T}(u - \tilde{u}) \le \sup _{\partial _{\mathcal {P}}\Omega _T}(u - \tilde{u}). $$

Since the reversed inequality holds trivially, this proves the claim. $\square$

### A Quantitative Bound on the Lipschitz Continuity

In this subsection, we establish a quantitative Lipschitz bound for parabolic minimizers of the gradient constraint problem under the $t-\textrm{BSC}_Q$.

#### Proposition 6.8

Let the assumptions of Theorem 1.3 be in force and $L\in (0,\infty ]$. Then, every parabolic minimizer $u\in \textrm{Lip}^x(\Omega _T, L)$ with $\partial _t u\in L^2(\Omega _T)$ in the sense of Definition 6.4 and Cauchy-Dirichlet boundary datum *g* satisfies the gradient bound

$$ \Vert \nabla u\Vert _{L^\infty (\Omega _T,\mathbb {R}^n)} \le C, $$

where *C* depends on $n, \varepsilon , R, {{\,\textrm{diam}\,}}(\Omega ), f, \nabla f, \Vert D^2f\Vert _{L^\infty (\mathbb {R}^n\setminus B_1)}, Q, [g]_{0,1;\Omega _T}, \mathsf Q$

#### Proof

Let $x_1\not = x_2$ two arbitrary points in $\Omega$ and $t\in (0,T)$. Define $y:=x_2-x_1$ and

$$\begin{aligned} u_y(x,t) := u(x+y,t), \qquad \text{ for } (x,t)\in \widetilde{\Omega }_T, \end{aligned}$$

where $\widetilde{\Omega }_T:=\{(x-y,t)\in \mathbb {R}^{n+1}: (x,t)\in \Omega _T\}$. Then, $u_y$ is a parabolic minimizer in $\widetilde{\Omega }_T$ in the class $\textrm{Lip}^x(\widetilde{\Omega }_T, L)$ in the sense of Definition 6.4. We denote the intersection of both cylinders by $(\Omega \cap \widetilde{\Omega })_T:=(\Omega \cap \widetilde{\Omega })\times (0,T)$. Lemma 6.5 ensures that both, *u* and $u_y$ are parabolic minimizers in $(\Omega \cap \widetilde{\Omega })_T$ in $\textrm{Lip}^x((\Omega \cap \widetilde{\Omega })_T, L)$. Therefore, from the maximum principle in Lemma 6.7 we conclude that there exists a boundary point $(x_o,t_o)\in \partial _{\mathcal {P}}\big ((\Omega \cap \widetilde{\Omega })_T\big )$ such that

$$\begin{aligned} |u(x_1,t)-u_y(x_1,t)| \le |u(x_o,t_o)-u_y(x_o,t_o)|. \end{aligned}$$

In view of the definition of $u_y$, this inequality yields

6.23 $$\begin{aligned} |u(x_1,t)-u(x_2,t)| \le |u(x_o,t_o)-u(x_o+y,t_o)|. \end{aligned}$$

Since $(x_o,t_o)\in \partial _{\mathcal {P}}\big ((\Omega \cap \widetilde{\Omega })_T\big )$, we either have $t_o=0$ or $x_o\in \partial (\Omega \cap \widetilde{\Omega })$. In the first case, we recall that $u(\cdot ,0)=g(\cdot ,0)$. Using the Lipschitz condition of *g*, we then obtain

$$\begin{aligned} \big |u(x_1,t)-u(x_2,t)\big | \le |g(x_o,0)-g(x_o+y,0)| \le \Vert \nabla g_o\Vert _{L^\infty (\Omega ,\mathbb {R}^n)}|y|. \end{aligned}$$

In the other case we know that one of the points $x_o$ or $x_o+y$ belongs to $\partial \Omega$. Without loss of generality we may assume $x_o\in \partial \Omega$. Since $u=g$ on the lateral boundary of $\Omega _T$, inequality (6.23) turns into

$$\begin{aligned} |u(x_1,t)-u(x_2,t)| \le |g(x_o,t_o)-u(x_o+y,t_o)|. \end{aligned}$$

By $v_{x_o}^\pm$ we denote the barrier functions constructed in Proposition 6.3 applied with $\alpha =\pm \alpha _o$. The barrier functions satisfy $v_{x_o}^\pm (x_o,t)=g(x_o,t)$ for any $t\in [0,T)$ and $v_{x_o}^-\le g\le v_{x_o}^+$ in $\Omega _T$. Moreover, $v_{x_o}^-$ is a sub-solution and since *f* is convex, it is also a parabolic sub-minimizer in the sense of Definition 6.4. Similarly, $v_{x_o}^+$ is a super-minimizer. Furthermore, $v_{x_o}^\pm$ are Lipschitz continuous with respect to the spatial variable with Lipschitz constant $\widetilde{Q}$ depending on $n, \varepsilon , R, {{\,\textrm{diam}\,}}(\Omega ), f, \nabla f$, $\Vert D^2f\Vert _{L^\infty (\mathbb {R}^n\setminus B_1)}, Q, [g]_{0,1;\Omega _T}, \mathsf Q$. Then, the maximum principle from Lemma 6.7 implies

$$\begin{aligned} v_{x_o}^-\le u\le v_{x_o}^+\qquad \text{ in } \Omega _T, \end{aligned}$$

so that

$$\begin{aligned} -\widetilde{Q}|y|&\le v_{x_o}^+(x_o,t)- v_{x_o}^+(x_o+y,t) \\&\le g(x_o,t)- u(x_o+y,t)\\&\le v_{x_o}^-(x_o,t)- v_{x_o}^-(x_o+y,t) \le \widetilde{Q}|y|. \end{aligned}$$

Therefore, we have

$$\begin{aligned} |u(x_1,t)-u(x_2,t)| \le \widetilde{Q}|y|. \end{aligned}$$

Joining both cases and recalling that $y=x_2-x_1$, we obtain

$$ |u(x_1,t)-u(x_2,t)| \le \max \big \{ \widetilde{Q}, \Vert \nabla g_o\Vert _{L^\infty (\Omega ,\mathbb {R}^n)}\big \} |x_1-x_2|, $$

which implies the claimed gradient bound. $\square$

### Proof of Theorem 1.3

In this section, we indicate how the gradient hypothesis $\Vert \nabla u\Vert _{L^\infty (\Omega ,\mathbb {R}^n)}\le L$ for a variational solution *u* of the gradient-constrained obstacle problem in $\textrm{Lip}^x_{g}(\Omega _{T}, L)$ can be removed, thereby obtaining the existence result from Theorem 1.3.

#### Proof of Theorem 1.3

Let $L>C$, where *C* denotes the constant from Proposition 6.8 depending only on $n, \varepsilon , R, {{\,\textrm{diam}\,}}(\Omega ){, f,} \nabla f, \Vert D^2f\Vert _{L^\infty (\mathbb {R}^n\setminus B_1)}, Q, [g]_{0,1;\Omega _T}, \mathsf Q$. Due to Proposition 5.1 there exists a unique variational solution $u\in \textrm{Lip}^x_g(\Omega _T,L)$ of the gradient constrained problem. The solution admits a weak time derivative $\partial _tu\in L^2(\Omega _T)$ and satisfies $u(0)=g_o$ in the $L^2(\Omega )$-sense. As pointed out at the beginning of § 6.3, *u* is also a parabolic minimizer in the sense of Definition 6.4. Proposition 6.8 ensures that the strict gradient bound

$$ \Vert \nabla u\Vert _{L^\infty (\Omega _T,\mathbb {R}^n)} \le C < L $$

holds. Therefore, it remains to prove that the variational inequality (1.6) satisfied by *u* actually holds for any comparison map $w\in \textrm{Lip}^x_{g}(\Omega _{T})$ with $\partial _t w\in L^2(\Omega _T)$. To this aim we consider $w\in \textrm{Lip}^x_{g}(\Omega _{T})$ and define

$$ v:= u+s(w-u)\qquad \text{ for } 0<s\ll 1. $$

Observe that *v* is an admissible comparison function in (6.21), since *v* coincides with *u* on the lateral boundary $\partial \Omega \times (0,T)$ and $\Vert \nabla v\Vert _{L^\infty (\Omega _T,\mathbb {R}^n)}<L$ for $s>0$ small enough. From (6.21) and the convexity of *f* we infer

$$\begin{aligned} \iint _{\Omega _T}f(\nabla u)\,\textrm{d}x\textrm{d}t&\le \iint _{\Omega _T}\big [s\partial _{t}u(w-u) + f\big ( (1-s)\nabla u+s\nabla w\big )\big ]\,\textrm{d}x\textrm{d}t\\&\le \iint _{\Omega _T}\big [s\partial _{t}u(w-u) + (1-s) f(\nabla u)+sf(\nabla w)\big ]\,\textrm{d}x\textrm{d}t. \end{aligned}$$

We re-absorb the second term of the right-hand side into the left and divide the result by $s>0$, so that

$$\begin{aligned} \iint _{\Omega _T}f(\nabla u)\,\textrm{d}x\textrm{d}t&\le \iint _{\Omega _T}\big [\partial _{t}u(w-u) + f(Dw)\big ]\,\textrm{d}x\textrm{d}t\\&= \iint _{\Omega _T}\big [\partial _{t}w(w-u)-\tfrac{1}{2}\partial _t|w-u|^2 + f(\nabla w)\big ]\,\textrm{d}x\textrm{d}t\\&= \iint _{\Omega _T}\big [\partial _{t}w(w-u) + f(\nabla w)\big ]\,\textrm{d}x\textrm{d}t\\&\quad + \tfrac{1}{2}\Vert w(0)-g_{o}\Vert _{L^{2}(\Omega )}^{2} - \tfrac{1}{2}\Vert (w-u)(T)\Vert _{L^{2}(\Omega )}^{2}. \end{aligned}$$

This shows that the variational inequality (1.6) holds for every comparison function $w\in \textrm{Lip}^x_{g}(\Omega _{T})$ that satisfies $\partial _tw\in L^2(\Omega _T)$. Therefore, *u* is a variational solution of the unconstrained problem in the sense of Definition 1.1. As before, the uniqueness can be deduced as in [9, Lemma 3.3] and the proof of Theorem 1.3 is complete. $\square$

## Regularity of Solutions and Proof of Theorem 1.4

Our aim in this section is to prove Theorem 1.4. By *u* we denote the unique variational solution from Theorem 1.3 satisfying

$$ \Vert \nabla u\Vert _{L^\infty (\Omega _T,\mathbb {R}^n)} \le C =: M. $$

Since *f* is assumed to be of class $C^1$ and $\nabla u$ is bounded, the associated Euler-Lagrange equation is well defined. Hence, *u* is a weak solution of the parabolic Cauchy-Dirichlet problem (1.3). From the Poincaré inequality for solutions to parabolic equations (cf. [8, Lemma 3.1]), we obtainfor any parabolic cylinder $Q_\varrho (z_o):=B_\varrho (x_o)\times (t_o-\varrho ^2,t_o+\rho ^2)\subset \Omega _T$ with $z_o=(x_o,t_o)\in \Omega _T$. A similar Poincaré inequality holds for parabolic cylinders with center $(x_o,t_o)\in \partial _{\mathcal {P}}\Omega _T$. This can be deduced as in [7], since $\Omega$ is convex and therefore also a Lipschitz domain. The parabolic version of Campanato’s characterization of Hölder continuity (with respect to the parabolic metric) by Da Prato [16] implies the Lipschitz continuity of *u* with respect to the parabolic metric, i.e. $u\in C^{0;1,\frac{1}{2}}(\overline{\Omega }_T)$. This finishes the proof of Theorem 1.4.

## Data Availability

Data sharing not applicable to this article as no datasets were generated or analyzed during the current study.

## References

[1] Akagi, G., Stefanelli, U.: Doubly nonlinear evolution equations as convex minimization. *SIAM J. Math. Anal.* **46**(3), 1922–1945, 2014

[2] Ambrosio, L., Gigli, N., Savaré, G.: *Gradient Flows in Metric Spaces and in the Space of Probability Measures. Lectures in Mathematics ETH Zürich*, 2nd edn. Birkhäuser Verlag, Basel, 2008

[3] Bögelein, V., Duzaar, F., Marcellini, P.: Parabolic systems with -growth: a variational approach. *Arch. Ration. Mech. Anal.* **210**(1), 219–267, 2013

[4] Bögelein, V., Duzaar, F., Marcellini, P.: Existence of evolutionary variational solutions via the calculus of variations. *J. Differ. Equ.* **256**(12), 3912–3942, 2014

[5] Bögelein, V., Duzaar, F., Marcellini, P.: A time dependent variational approach to image restoration. *SIAM J. Imaging Sci.* **8**(2), 968–1006, 2015

[6] Bögelein, V., Duzaar, F., Marcellini, P., Signoriello, S.: Parabolic equations and the bounded slope condition. *Ann. Inst. H. Poincaré C Anal. Non Linéaire* **34**(2), 355–379, 2017

[7] Bögelein, V., Duzaar, F., Mingione, G.: The boundary regularity of nonlinear parabolic systems I. *Ann. Inst. H. Poincaré Anal. Non Linéaire* **27**(1), 201–255, 2010

[8] Bögelein, V., Duzaar, F., Mingione, G.: The regularity of general parabolic systems with degenerate diffusion. *Mem. Am. Math. Soc.* **221**(1041), vi+143, 2013

[9] Bögelein, V., Duzaar, F., Scheven, C.: The obstacle problem for parabolic minimizers. *J. Evol. Equ.* **17**(4), 1273–1310, 2017

[10] Bousquet, P.: On the lower bounded slope condition. *J. Convex Anal.* **14**(1), 119–136, 2007

[11] Bögelein, V., Stanin, T.: The one-sided bounded slope condition in evolution problems. *Ann. Mat. Pura Appl.* **199**(4), 573–587, 2020

[12] Bousquet, P.: Boundary continuity of solutions to a basic problem in the calculus of variations. *Adv. Calc. Var.* **3**(1), 1–27, 2010

[13] Cellina, A.: On the bounded slope condition and the validity of the Euler Lagrange equation. *SIAM J. Control Optim.* **40**(4), 1270–1279, 2002

[14] Cellina, A.: Uniqueness and comparison results for functionals depending on and on . *SIAM J. Optim.* **18**(3), 711–716, 2007

[15] Clarke, F.: Continuity of solutions to a basic problem in the calculus of variations. *Ann. Sc. Norm. Super. Pisa Cl. Sci. (5)* **4**(3), 511–530, 2005

[16] Da Prato, G.: Spazi e loro proprietà. *Ann. Mat. Pura Appl IV.* **69**, 383–392, 1965

[17] De Giorgi, E.: Conjectures concerning some evolution problems. (Italian) A celebration of John F. Nash, Jr. *Duke Math. J.* **81**(2), 255–268, 1996

[18] Ekeland, I., Témam, R.: Convex analysis and variational problems, Society for Industrial and Applied Mathematics (SIAM), Philadelphia, PA, ISBN 0-89871-450-8 1999.

[19] Fiaschi, A., Treu, G.: The bounded slope condition for functionals depending on , , and . *SIAM J. Control Optim.* **50**(2), 991–1011, 2012

[20] Giannetti, F., Treu, G.: On the Lipschitz regularity for minima of functionals depending on , , and under the bounded slope condition. *SIAM J. Control Optim.* **60**(3), 1347–1364, 2022

[21] Giannetti, F., Treu, G.: Local Lipschitz continuity of the minimizers of nonuniformly convex functionals under the Lower Bounded Slope Condition, Calc. Var. Partial Differ. Equat. 10.1007/s00526-026-03303-7, arXiv https://arxiv.org/abs/2504.11594

[22] Giusti, E.: *Direct Methods in the Calculus of Variations*, World Scientific, Singapore and River Edge and NJ ISBN 9789812380432 2003

[23] Hardt, R., Zhou, X.: An evolution problem for linear growth functionals. *Commun. Partial Differ. Equ.* **19**(11–12), 1879–1907, 1994

[24] Haar, A.: Über das Plateausche Problem. *Math. Ann.* **97**(1), 124–158, 1927

[25] Hartman, P., Nirenberg, L.: On spherical image maps whose Jacobians do not change sign. *Am. J. Math.* **81**, 901–920, 1959

[26] Hartman, P., Stampacchia, G.: On some non-linear elliptic differential-functional equations. *Acta Math.* **115**, 271–310, 1966

[27] Hilbert, D.: Über das Dirichletsche Prinzip. *Math. Ann.* **59**(1–2), 161–186, 1904

[28] Ilmanen, T.: Elliptic regularization and partial regularity for motion by mean curvature. *Mem. Am. Math. Soc.* **108**(520), x+90, 1994

[29] Kinnunen, J., Lindqvist, P.: Pointwise behaviour of semicontinuous supersolutions to a quasilinear parabolic equation. *Ann. Mat. Pura Appl. (4)* **185**(3), 411–435, 2006

[30] Lichnewsky, A., Temam, R.M.: Pseudosolutions of the time-dependent minimal surface problem. *J. Differ. Equ.* **30**(3), 340–364, 1978

[31] Mariconda, C., Treu, G.: Existence and Lipschitz regularity for minima. *Proc. Am. Math. Soc.* **130**(2), 395–404, 2002

[32] Mariconda, C., Treu, G.: Lipschitz regularity for minima without strict convexity of the Lagrangian. *J. Differ. Equ.* **243**(2), 388–413, 2007

[33] Mariconda, C., Treu, G.: Hölder regularity for a classical problem of the calculus of variations. *Adv. Calc. Var.* **2**(4), 311–320, 2009

[34] Mascolo, E., Schianchi, E.: Existence theorems for nonconvex problems. *J. Math. Pures Appl. (9)* **62**(3), 349–359, 1983

[35] Mielke, A., Stefanelli, U.: Weighted energy-dissipation functionals for gradient flows. *ESAIM Control Optim. Calc. Var.* **17**(1), 52–85, 2011

[36] Miranda, M.: Un teorema di esistenza e unicità per il problema dell’area minima in n variabili. *Ann. Scuola Norm. Sup. Pisa* **19**(3), 233–249, 1965

[37] Rainer, R., Siltakoski, J., Stanin, T.: An evolutionary Haar-Rado type theorem. *Manuscr. Math.* **168**(1–2), 65–88, 2022

[38] Schätzler, L.: Existence of variational solutions for time dependent integrands via minimizing movements. *Analysis* **37**(4), 199–222, 2017

[39] Serra, E., Tilli, P.: Nonlinear wave equations as limits of convex minimization problems: proof of a conjecture by De Giorgi. *Ann. Math. (2)* **175**(3), 1551–1574, 2012

[40] Stampacchia, G.: On some regular multiple integral problems in the calculus of variations. *Commun. Pure Appl. Math.* **16**, 383–421, 1963

[41] Stanin, T.: Global continuity of variational solutions weakening the one-sided bounded slope condition. *Forum Math.* **33**(5), 1237–1260, 2021

[42] Stefanelli, U.: The De Giorgi conjecture on elliptic regularization. *Math. Models Methods Appl. Sci.* **21**(6), 1377–1394, 2011

[43] Wieser, W.: Parabolic Q-minima and minimal solutions to variational flow. *Manuscr. Math.* **59**(1), 63–107, 1987

[44] Zhou, X.: An evolution problem for plastic antiplanar shear. *Appl. Math. Optim.* **25**(3), 263–285, 1992

